# *In Vivo* Efficacy of Artemether-Lumefantrine and Chloroquine against *Plasmodium vivax:* A Randomized Open Label Trial in Central Ethiopia

**DOI:** 10.1371/journal.pone.0063433

**Published:** 2013-05-22

**Authors:** Jimee Hwang, Bereket Hailegiorgis Alemayehu, Richard Reithinger, Samuel Girma Tekleyohannes, Takele Teshi, Sintayehu Gebresillasie Birhanu, Leykun Demeke, David Hoos, Zenebe Melaku, Moges Kassa, Daddi Jima, Joseph L. Malone, Henry Nettey, Michael Green, Amanda Poe, Sheila Akinyi, Venkatachalam Udhayakumar, S. Patrick Kachur, Scott Filler

**Affiliations:** 1 Malaria Branch, Centers for Disease Control and Prevention, Atlanta, Georgia, United States of America; 2 Global Health Group, University of California San Francisco, San Francisco, California, United States of America; 3 ICAP-Columbia University, New York, New York, United States of America; 4 U.S. Agency for International Development, Addis Ababa, Ethiopia; 5 Research Triangle Institute, Washington, DC, United States of America; 6 ICAP-Columbia University, Addis Ababa, Ethiopia; 7 Ethiopian Health and Nutrition Research Institute, Addis Ababa, Ethiopia; 8 Federal Ministry of Health, Addis Ababa, Ethiopia; 9 School of Pharmacy, University of Ghana, Legon, Ghana; 10 Atlanta Research and Education Foundation, Decatur, Georgia, United States of America; 11 The Global Fund to Fight AIDS, Tuberculosis and Malaria, Geneva, Switzerland; Menzies School of Health Research, Australia

## Abstract

**Background:**

*In vivo* efficacy assessments of antimalarials are essential for ensuring effective case management. In Ethiopia, chloroquine (CQ) without primaquine is the first-line treatment for *Plasmodium vivax* in malarious areas, but artemether-lumefantrine (AL) is also commonly used.

**Methods and Findings:**

In 2009, we conducted a 42-day efficacy study of AL or CQ for *P. vivax* in Oromia Regional State, Ethiopia. Individuals with *P. vivax* monoinfection were enrolled. Primary endpoint was day 28 cure rate. In patients with recurrent parasitemia, drug level and genotyping using microsatellite markers were assessed. Using survival analysis, uncorrected patient cure rates at day 28 were 75.7% (95% confidence interval (CI) 66.8–82.5) for AL and 90.8% (95% CI 83.6–94.9) for CQ. During the 42 days of follow-up, 41.6% (47/113) of patients in the AL arm and 31.8% (34/107) in the CQ arm presented with recurrent *P. vivax* infection, with the median number of days to recurrence of 28 compared to 35 days in the AL and CQ arm, respectively. Using microsatellite markers to reclassify recurrent parasitemias with a different genotype as non-treatment failures, day 28 cure rates were genotype adjusted to 91.1% (95% CI 84.1–95.1) for AL and to 97.2% (91.6–99.1) for CQ. Three patients (2.8%) with recurrent parasitemia by day 28 in the CQ arm were noted to have drug levels above 100 ng/ml.

**Conclusions:**

In the short term, both AL and CQ were effective and well-tolerated for *P. vivax* malaria, but high rates of recurrent parasitemia were noted with both drugs. CQ provided longer post-treatment prophylaxis than AL, resulting in delayed recurrence of parasitemia. Although the current policy of species-specific treatment can be maintained for Ethiopia, the co-administration of primaquine for treatment of *P. vivax* malaria needs to be urgently considered to prevent relapse infections.

**Trial Registration:**

ClinicalTrials.gov NCT01052584

## Introduction

*Plasmodium vivax* transmission is more widely distributed globally than *P. falciparum,* with 2.85 billion people living at risk of its infection [1]. In Ethiopia, malaria is a leading cause of morbidity and mortality, with 14% of all outpatient visits and 9% of hospital admissions being due to malaria in 2009–2010 [2]. Unlike much of Africa, both *P. falciparum* and *P. vivax* substantially contribute to malaria morbidity in Ethiopia, in relative proportions of approximately 60% and 40% [3], [4], [5], respectively, although their relative proportions vary both temporally and spatially.

With wide-spread *P. falciparum* resistance to chloroquine (CQ) [6] and sulphadoxine-pyrimethamine (SP) [7], [8], in 2004, the Ethiopian Federal Ministry of Health (FMOH) adopted artemether-lumefantrine (AL) as first-line treatment of uncomplicated *P. falciparum* malaria and of mixed infections with both *P. falciparum* and *P. vivax* [9]. Although CQ remains the first-line treatment for *P. vivax* malaria in Ethiopia, AL is used widely for all cases of mixed infections and clinical malaria where laboratory diagnostics to determine the specific malaria species are not available or not done.

Following the first report of CQ-resistant *P. vivax* in 1989 from Papua New Guinea [10], decreased CQ susceptibility have been reported from Indonesia [11], [12], [13], [14], Papua New Guinea [15], Burma [16], Vietnam [17], [18], Turkey [19], [20], Colombia [21], and Brazil [22]. Overall, CQ-resistant *P. vivax* has remained rare in Africa. In 1996, Ethiopia published its first report of CQ resistance, with 2% (5/255) of study patients on CQ with persistent parasitemia on day 7 [23]. Subsequent reports from Ethiopia documented presence of CQ-resistant *P. vivax*, but at levels not exceeding 5% [24], [25], [26].

After adopting AL as first-line therapy for uncomplicated *P. falciparum* malaria, mixed infections, and clinically diagnosed malaria, the Ethiopian FMOH scaled up an ambitious plan to provide universal access to prompt malaria diagnosis and treatment –as recommended by the World Health Organization (WHO) [27]– through a network of 15,000 community-level health posts [28]. With the use of multi-species malaria rapid diagnostic tests (RDTs) to distinguish *P. falciparum* from *P. vivax* infection and expansion of quality malaria microscopy diagnostic capacity, administration of CQ increased in Ethiopia since 2004 in parallel with increased numbers of laboratory confirmed *P. vivax* mono-infections. Since both AL and CQ without primaquine are widely used according to FMOH guidelines for *P. vivax* infections in malarious areas, continuously monitoring *in vivo* drug efficacy to these medications for *P. vivax* malaria is recommended by WHO [29], [30] to allow for sufficient time to explore alternatives and change national policy when efficacies are observed to decline.

The objectives of the study reported here were (i) to assess the *in vivo* efficacy of AL and CQ for treatment of *P. vivax* for 28 days, and (ii) to assess secondary outcomes of day 42 efficacy, hematologic response, and fever and parasite clearance rates.

## Methods

The protocol of this trial and supporting CONSORT checklist are available as supporting information (see Protocol S1 and Checklist S1).

### Study Site and Enrollment

This randomized, open-label study was conducted at two established FMOH health facilities, the Bishoftu Malaria Clinic and the Bulbula Health Center, in Oromia Regional State, Ethiopia, from October 2009 to January 2010. *P. falciparum* and *P. vivax* are endemic in the catchment areas of both sites, and the study was conducted during the peak malaria transmission season which occurs between September to December. Detailed study site information has been previously reported along with the results of the *P. falciparum* treatment outcomes [31].

### Patients

Patients with *P. vivax* mono-infection, with parasitemia density >250 asexual forms per µl were enrolled. Additional inclusion criteria included age >6 months, weight >5 kg, and axillary temperature ≥37.5°C or history of fever in the past 48 hours. Only patients living within 20 km of the facilities were enrolled in the study to facilitate follow-up. Exclusion criteria included detection of any *Plasmodium* sp. infection besides *P. vivax* mono-infection, any signs or symptoms of severe illness, severe malnutrition, severe anemia (hemoglobin<5 g/dL), known hypersensitivity to AL or CQ, pregnancy, breastfeeding, or presence of another infection or major co-morbid conditions by history.

The study received ethical clearance from the U.S. Centers for Disease Control and Prevention, Columbia University, and the Ethiopian Public Health Association. Written informed consent was obtained from adult patients and guardians of enrolled children. Written assent was also obtained from children 7–17 years of age.

### Clinical and Laboratory Procedures

To assess AL and CQ therapeutic efficacy for uncomplicated *P. vivax* mono-infection based on clinical and parasitological parameters, we conducted a prospective, 42-day, randomized, open-label *in vivo* efficacy assessment according to the 2003 and 2009 WHO *in vivo* protocol for measuring antimalarial drug efficacy for areas of low to moderate malaria transmission [29], [30]. At the Bishoftu Malaria Clinic, blood films were taken at initial presentation for all patients (as routinely done). Once a positive *P. vivax* infection was identified by microscopy, the patient was screened for study eligibility criteria. At Bulbula Health Center, all patients attending the outpatient ward were initially clinically evaluated by the health facility staff; those with fever or a history of fever were referred for malaria microscopy testing (as routinely done). Once the patient was noted to have microscopy-confirmed *P. vivax* infection, the study staff screened the patient for inclusion in the study.

After meeting the inclusion criteria and consenting to study enrollment, all patients underwent hemoglobin testing (Hemocue Hb 201+, Angelholm, Sweden) and filter paper blood spot collection. All women aged 13–49 years underwent a urine pregnancy test and those testing positive were excluded from the study. Giemsa-stained thick and thin blood films were prepared according to standard procedures [29] and read by two independent microscopists blinded to the other read and the assigned treatment arms. Parasite density was estimated based on the number of asexual parasites observed against 200 leukocytes assuming 8,000 leukocytes per µl. Slides with parasite densities differing by more than 20% between microscopists were reassessed by a third microscopist, with the third reading considered final. Smears were determined to be negative only after examining 100 high power oil immersion fields.

Patients were enrolled, interviewed and examined by the study clinician. The patients were randomized to standard dosages of oral AL (Coartem®, Novartis Pharma, Basel, Switzerland) or CQ (Aralen®, Sanofi-Aventis, US) based on weight according to national policy [9]. Randomization sequence was computer generated by the principal investigator and kept in sealed envelopes. Clinicians enrolled the patients and nurses assigned the patients sequentially according to the sealed envelopes. Artemether-lumefantrine was packaged in fixed-dose combination tablets, each containing 20 mg of artemether and 120 mg of lumefantrine. Artemether-lumefantrine was administered according to the package insert twice daily for three days: patients weighing 5–14 kg, 15–24 kg, 25–34 kg, and ≥35 kg were given one tablet, two tablets, three tablets, and four tablets at each of six dosing intervals, respectively. The initial and each morning dose were directly observed by the study staff and given with milk biscuits to increase absorption [32], [33]. A total of 25 mg base per kg over three days (10 mg base/kg on day 1 and 2, and 5 mg base/kg on day 3) of CQ was administered based on weight using 10 mg/ml syrup or dividing 500 mg tablets accordingly. All patients were monitored for 60 minutes; a full or half a dose was re-administered if the patient vomited the drug within 30 or 31–60 minutes, respectively. If the patient vomited again, he or she was referred to the hospital and withdrawn from the study. Patients were instructed to take the evening dose with food. On follow-up days 1–3, patients taking AL were asked if the drug was taken properly the previous night. In order to facilitate twice daily dosing of AL, all patients presenting after 1500 hrs were excluded from the study. Antipyretics were given for temperatures >39°C as per national policy [9]. Ferrous sulfate, folate, and mebendazole (if aged >1 year) were given to all children with hemoglobin<10 mg/dl as per Integrated Management of Childhood Illness guidelines [34]. Quinine (10 mg mg/kg every 8 hours for 7 days), the second-line treatment as per national policy [9], was administered as rescue therapy.

### Follow-up

Patients were followed-up for 42 days and asked to return on days 1–3, 7, 14, 21, 28, 35, and 42 post-treatment, as well as any other interim day if ill. The study site facilities were open from 0800 to 1800 hrs, and after hours care was also available. Standardized follow-up included documentation of history-taking to elicit symptoms, adverse events, and any concomitant therapy, and physical examination including axillary temperature measurement. All symptoms elicited during follow-up that were not present on day 0 were classified as an adverse event. For all adverse events, severity, duration, outcome and its likely relatedness to the administered drug were assessed by the clinicians. Adverse events were reported for all enrolled patients. Finger pricks for follow-up blood films were taken on scheduled days 2, 3, 7, 14, 21, 28, 35, 42, and at any unscheduled visit. Hemoglobin was measured weekly and filter papers were collected on day 7 for drug level testing and on any day of recurrent parasitemia (after day 3) for CQ level and parasitemia molecular testing. All filter papers were dried and stored in plastic storage bags with desiccant and humidity indicators. The filter papers for drug level and molecular testing were sent to the U.S. Centers for Disease Control and Prevention in Atlanta, USA. All additional sample investigations such as the drug level and molecular testing were performed by individuals who were blinded to the treatment allocation.

### Outcomes

Efficacy was assessed by clinical and parasitological outcomes using WHO definitions, with a 42-day follow-up period [30], with treatment failure defined as 1) clinical deterioration due to *P. vivax* illness requiring hospitalization in presence of parasitemia; or 2) presence of parasitemia and axillary temperature ≥37.5°C anytime between days 3 and 28/42; or 3) presence of parasitemia on any day between days 7 and 28/42, irrespective of clinical conditions. If no failure was recorded, outcome was classified as a treatment success.

### Laboratory Analysis

To refine the identification of recrudescences, three to four drops of blood were collected on filter paper at day 0 prior to treatment, day 7, and on any day of recurrent parasitemia. Blood spots collected on the day of enrollment (day 0) and on the day of failure were used for molecular analysis to determine the genetic identity of the parasites. Blood spots collected on day 7 or day of treatment failure were tested for lumefantrine and CQ drug levels, respectively.

#### Microsatellite characterization

Eight of the eleven microsatellite markers described by Imwong et al. [35] were chosen for the final analysis in this study. Although marker 2208 amplified only in a subset of samples, it was included in the final analysis, because allelic difference in this locus was useful to differentiate strains. The published PCR protocol [35] was slightly modified by using a commercial PCR MasterMix (Promega, Madison, WI). Length variation in fluorescently-labeled PCR products was determined on an Applied Biosystems Prism 3130×l Avant Genetic Analyzer and the data analyzed using GeneMapper v4.0 (all Applied Biosystems, Foster City, CA). When the sizes of alleles at a microsatellite marker differed by >2 base pairs, they were considered different alleles.

The genetic variation for each microsatellite locus was measured by calculating the expected heterozygosity (*H_e_*) and number of alleles per microsatellite locus (*A*). *H_e_* was calculated for each locus as *H_e_* = [*n*/(*n*–1)][1–∑*p_i_^2^*], where *n* is the number of isolates sampled and *p_i_* is the frequency of the *i*th allele. The sampling variance for *H_e_* was calculated as 2(*n*–1)/*n* ^3^[2(*n*–2)[∑*p_i_* ^3^–(∑*p_i_* ^2^)^2^]] [36]. Arlequin ver 3.01 (University of Bern, Switzerland) was used to compute *H_e_* [37], and the Excel Microsatellite Toolkit was used to format data for use in Arlequin [38]. When parasites on day 0 and the day of failure were different from each other at one or more loci in their microsatellite profile, then these parasites were considered genetically non-identical and regarded as different strains for the purpose of this study. Recurrent parasitemias with identical genotypes from day 0 were classified as recrudescences (true drug resistance) although this group could include homologous relapses. The PCR-adjusted cure rate (the sum of cases with no recurrent parasites and recurrent with different genotypes) attempts to reduce the contribution from new infections and heterologous relapses representing a refined ‘minimum’ cure rate.

#### Drug level testing

Lumefantrine levels from day 7 dried blood spots were assessed in all cases of AL recurrent parasitemia. Lumefantrine levels in capillary whole blood were measured from dried blood spots using a modified high-performance liquid chromatographic method [30]. Due to logistical constraints, it was impossible to treat the filter papers with tartaric acid prior to blood application as suggested by Blessborn et al. [30], resulting in a lower recovery efficiency and a higher limit of quantification of 212 ng/ml.

Similarly, CQ levels from dried blood spots collected on the day of failure were assessed in all cases of CQ treatment failure. Chloroquine and its metabolite, desethylchloroquine (DCQ), were measured using a high-performance liquid chromatographic method as described by Patchen et al. [39]. The limit of detection for both CQ and DCQ was 5 ng/ml. Levels<5 ng/ml were designated as below detection.

### Statistical Analysis

Primary efficacy outcome was day 28 cure rates. Secondary outcomes included day 42 cure rates, genotype adjusted minimum cure rates, hematologic outcomes, and fever and parasite clearance. With treatment failure of AL and CQ for *P. vivax* being reported at ≤5%, 10% was chosen as the estimated therapeutic failure rate. Assuming an estimated cure rate of 90% and a 20% loss to follow-up at 42 days, a sample size of 120 subjects per arm was calculated to result in a 95% exact binomial confidence interval from 82.4%–95.1%. The study was not powered to estimate differences in efficacy across treatment arms or between sites.

All data were entered into both a Microsoft Access (Microsoft, Redmond, WA, USA) study database and the Microsoft Excel (Microsoft, Redmond, WA, USA) standard database developed by WHO [30], and analyzed using SAS 9.2 (SAS Institute, Cary, NC, USA). Data were double-entered and any discrepancies resolved by referring to the original paper form. The cumulative risk of failure was computed for 28 and 42 days of follow-up using a modified intention to treat Kaplan-Meier survival analysis, the most appropriate way to estimate failure rates: parasitological recurrence is classified as treatment failure on the day it occurred [40], where lost to follow-up, withdrawals, and parasitemia with a different species are censored on the last day of follow-up. For survival analyses adjusting for microsatellite results, isolates with different genotype for primary and recurrent parasitemias were censored on the last day of recurrence. Comparisons between groups were made using a χ^2^ test or Fisher’s exact test for categorical variables and the Student’s t-test or Wilcoxon-Mann-Whitney test for continuous variables. A two-sided p-value<0.05 was considered statistically significant.

## Results

### Patients

**Figure 1:**
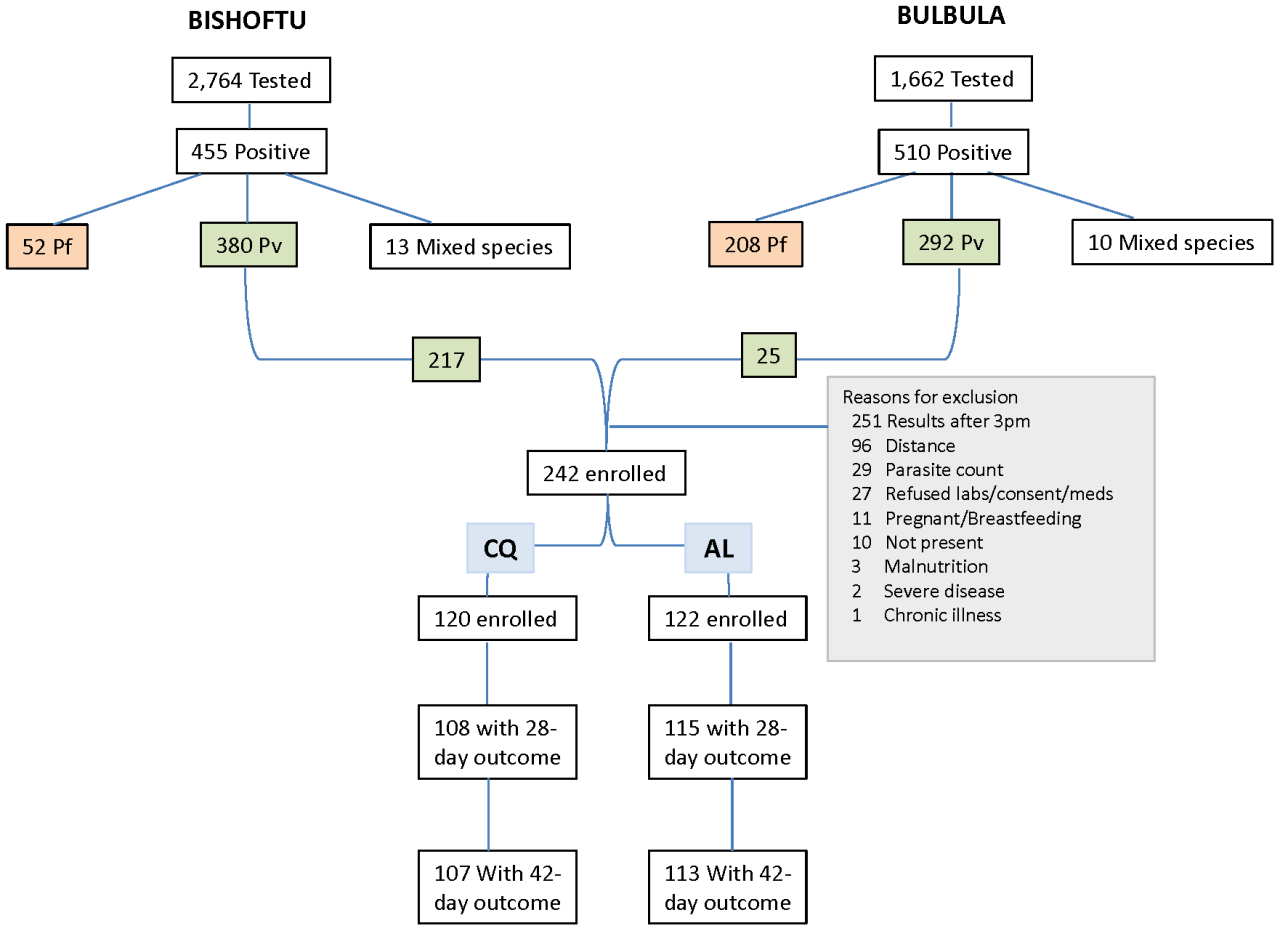
Trial Profile. Screening, Enrollment, and Follow-up of Patients.

Of the 4,426 patients tested from the two study sites, 961 were positive for malaria parasites from October 21 to November 30, 2009. The last date of follow-up occurred on January 11, 2010. Of 672 patients with confirmed *P. vivax* mono-infections, 242 (217 from Bishoftu and 25 from Bulbula) were enrolled for the *in vivo* drug efficacy study, with 122 and 120 patients randomized to receive AL or CQ, respectively (Figure 1). At the Bishoftu Malaria Clinic where all presenting patients received microscopy testing, the slide positivity rate was 16.5% (455/2,764), of which *P. vivax* mono-infection accounted for 83.5% (380/455). At Bulbula Health Center where only suspected malaria cases received microscopy, the slide positivity rate was 30.7% (510/1,662) of which *P. vivax* mono-infection accounted for 57.3% (292/510). The majority of excluded patients presented late in the afternoon and was therefore not enrolled to ensure appropriate timing of the second AL dose.

Although drug assignment was randomized, there was a statistically significant baseline difference in age (p = 0.03), weight (p = 0.04), height (p = 0.05), and ownership of bed nets with fewer patients in the AL drug arm owning one (p = 0.02) (Table 1). The other characteristics did not differ significantly, but the study was not powered to compare the drug arms.

**Table 1:** Baseline characteristics of enrolled patients by treatment group.

	Artemether-lumefantrine	Chloroquine
Characteristic	N = 122	N = 120
Female, n (%)	46 (37.7%)	38 (31.7%)
Median age, years (range)	11.5 (1–70)	18 (1–65)
Median height, cm (range)	144 (70–183)	159 (75–190)
Median weight, kg (range)	30 (6–79)	48.5 (8–76)
Median temperature, °C (range)	37.0 (35–40.5)	37.0 (35–40.3)
Median hemoglobin, g/dL (range)	13.0 (5.7–17.6)	13.5 (6.9–18)
Geometric Mean Parasite Density (range)	3179 (280–28,400)	2561 (280–42,000)
Owns a bednet, n (%)	51 (41.8%)	68 (56.7%)
IRS in past 12 months, n (%)	64 (52.5%)	72 (60%)

### Treatment Outcomes

Follow-up was completed for 114 patients to day 28 and 113 to day 42 in the AL arm and 108 patients to day 28 and 107 to day 42 in the CQ arm (Figure 1). Six of 122 patients were lost to follow-up and three withdrew from the study in the AL arm, whereas, ten of 120 patients were lost to follow-up and three withdrew from the CQ arm (Table 2). The total attrition rate was 8.0% for the AL arm and 12.1% in the CQ arm.

**Table 2:** Treatment outcomes after day 28 and 42 of follow-up by treatment arm.

	Artemether-lumefantrine (n = 122)		Chloroquine (n = 120)	
Outcome	Day 28	Day 42	Day 28	Day 42
No treatment outcome, n (%)	8 (6.6)	9 (8.0)	12 (11.1)	13 (12.1)
Lost to follow-up	6	6	9	10
Protocol violation	2	3	3	3
Recurrent parasitemia, n (%)	28 (24.3)	47 (41.6)	10 (9.3)	34 (31.8)
Bishoftu, n/N (%)	28/115 (24.4)	46/115 (40)	8/102 (7.8)	30/102 (29.4)
Bulbula, n/N (%)	0/7 (0)	1/7 (14.3)	2/18 (11.1)	4/18 (22.2)
Cure rate–Survival analysis, % (95% CI)	75.7 (66.8–82.5)	58.8 (49.2–67.2)	90.8 (83.6–94.9)	68.4 (58.7–76.3)
Bishoftu, % (95% CI)	74.6 (65.4–81.7)	57.9 (48.1–66.5)	91.2 (83.1–95.5)	66.7 (56.0–75.4)
Bulbula, % (95% CI)	100	80.0 (20.4–96.9)	88.9 (62.4–97.1)	77.0 (49.7–90.7)
Genotype of recurrent parasitemias, n (%)				
Different*	18 (64.3)	31 (66.0)	7 (70.0)	18 (52.9)
Identical	10 (35.7)	16 (34.0)	3 (30.0)	16 (47.1)
Minimum cure rate (adjusted for genotype*)–Survival analysis,% (95% CI)	91.1 (84.1–95.1)	84.3 (75.6–90.1)	97.2 (91.6–99.1)	83.6 (74.6–89.7)

* Greater than 2 base pair difference detected in at least one out of eight markers between the Day 0 (pre-treatment) and day of failure samples.

CI- confidence interval.

Treatment with both AL and CQ resulted in rapid clearance of parasites and reduction of fever. Whereas in the AL arm all patients had cleared their peripheral parasitemia by day 2, 6.0% (7/117) of the patients in the CQ arm had peripheral parasitemia by day 2 and 0.9% (1/115) remained positive on day 3 (Table 3). The difference in day 2 positivity was statistically significant (p = .006). Of those with persistent peripheral parasitemia on day 2, one was lost to follow-up, but the remaining with outcome data did not develop recurrent parasitemia.

**Table 3:** Secondary outcomes by treatment arm.

Outcome	Artemether-lumefantrine	Chloroquine
Fever, n/N (%)*		
Day 1	76/122 (62.3)	66/118 (55.9)
Day 2	31/121 (25.6)	26/117 (22.2)
Day 3	13/121 (10.7)	11/115 (9.6)
Parasitemia, n/N (%)		
Day 2	0/121 (0)	7/117 (6.0)
Day 3	0/121 (0)	1/115 (0.9)
Days to recurrence, median (Range)	28 (21–42)	35 (21–42)
Median Hemoglobin g/dL, mean (SD)		
Day 7	12.5 (1.7)	13.2 (2.0)
Day 28	13.7 (1.7)	14.0 (1.7)
Day 42	13.9 (1.5)	14.2 (1.8)
Median difference in hemoglobin from day 0 to day 7	0 (0.1)	−0.02 (0.1)
Median difference in hemoglobin from day 0 to day 42	0.06 (0.18)	0.06 (0.17)

* Subjective fever or axillary temperature ≥37.5°C; CI- confidence interval.

**Figure 2:**
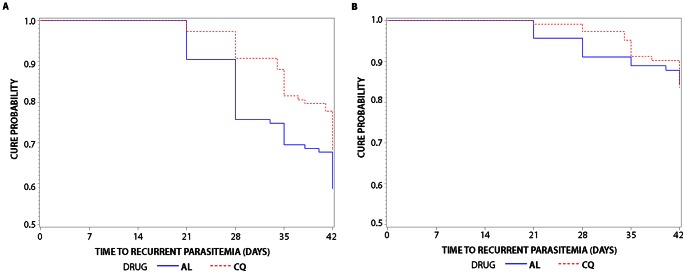
Kaplan-Meier Survival Curves for time to recurrent parasitemia during the 42 days of follow-up. (A) Time to any recurrent *P. vivax* parasitemia by drug arm; (B) Time to only recurrent *P. vivax* parasitemia with the same genotype by drug arm. AL: artemether-lumefantrine; CQ: chloroquine.

In the AL arm, 28 patients (24.3%) presented with recurrent parasitemia by day 28, and 47 (41.6%) by day 42 (Table 2). In the CQ arm, ten patients (9.3%) presented with recurrent parasitemia by day 28 and 34 (31.8%) by day 42 (Table 2). The median time to appearance of recurrent parasitemia was 28 days (range 21–42) in the AL arm and 35 days (range 21–42) in the CQ arm (Table 3). The earliest day of recurrent parasitemia was day 21 for both treatment arms. The PCR uncorrected cure rates at day 28 and 42 in the AL arm were 75.7% (95% CI 66.8–82.5) and 58.8% (95% CI 49.2–67.2), respectively (Table 2 and Figure 2A).The uncorrected cure rates at day 28 and 42 in the CQ arm were 90.8% (95% CI 83.6–94.9) and 68.4% (95% CI 58.7–76.3), respectively (Table 2 and Figure 2A). The day 28 PCR uncorrected cure rate for the CQ arm was higher than the AL arm, which was statistically significant (p = 0.003). The varying proportion of patients presenting with recurrent parasitemia and the cure rates by study site are shown in Table 2 which suggests higher recurrent parasitemia rates in Bishoftu. However, with such small number of patients from Bulbula the confidence intervals are too wide to draw any meaningful conclusions.

In the AL arm, the median age for those patients with or without recurrent parasitemia was seven and 17.5 years of age, respectively (p<0.0001). In the CQ arm, the median age for those patients with or without recurrent parasitemia was nine and 20 years of age, respectively (p<0.0001). In both treatment arms, younger age was associated with treatment failure. In the CQ arm, those with recurrent parasitemia had a higher median parasite density (4780 parasites/µL) compared to those without recurrent parasitemia (2120 parasites/µL) which was statistically significant (p = 0.01).

### Laboratory Outcomes

#### Hematologic response

In the AL arm, median hemoglobin increased from 13.0 g/dL (standard deviation (SD) 2.3) on day 0 to 13.9 g/dL (SD 1.5) on day 42 (Table 3). In the CQ arm, median hemoglobin increased from 13.5 g/dL (SD 2.5) on day 0 to 14.2 g/dL (SD 1.8) on day 42. The difference in median hemoglobin levels between day 42 and 0 was 0.06 g/dL (SD 0.18) in the AL arm and 0.06 g/dL (SD 0.17) in the CQ arm.

#### Microsatellite analysis of paired samples

Day 0 and day of recurrent parasitemia samples were analyzed for *P. vivax* strain identity using the size polymorphisms in eight microsatellite loci as reported in Table 4. For the AL arm, 47 pairs were analyzed and 16 pairs were identical at all loci (Table 2). For the CQ arm, 34 pairs were analyzed and 16 pairs were identical at all loci. It is worth noting that most of the microsatellite markers used in this study (except marker 2208) were highly diverse as indicated by high *H_E_* values and several allelic forms existed for all the markers used in this study (Table 4). This data provide further support that these combination of markers can reliably differentiate genetic relatedness of parasite populations in this study. Since paired samples identified as different strains are not true drug resistant infections, the PCR adjusted cure rates for the AL arm at day 28 were refined from 75.7% to 91.1% (95% CI 84.1–95.1) and for the CQ arm from 90.8% to 97.2% (95% CI 91.6–99.1) (Table 2). The day 42 PCR adjusted cure rates were 84.3% (95% CI 75.6–90.1) for the AL arm and 83.6% (95% CI 74.6–89.7) for the CQ arm (Table 2 and Figure 2B). Overall, 49 pairs were different in at least one locus and they were considered different strains (Table S1).

**Table 4:** Microsatellite diversity for eight potential markers for *P. vivax* by pre- and post-treatment.

Microsatellite marker name	14.185	12.335	7.67	2208	8.332	6.34	10.29	3.35
Microsatellite	v23	v24	v25	v27	v29	v30	v32	v33
**Allelic size range, bp**								
** D0**	263–273	156–196	100–125	158–169	216–252	136–173	116–130	112–128
** DF**	263–283	155–196	100–125	159–169	231–252	135–173	115–134	112–128
**No. of alleles**								
** D0**	8	11	18	6	12	16	10	11
** DF**	7	15	18	4	13	18	11	13
**PCR positivity**								
** D0**	82	79	73	39	80	81	82	82
** DF**	82	79	67	25	81	81	80	81
**MOI**								
** D0**	0	2	13	0	3	3	0	8
** DF**	1	2	11	0	3	1	0	1
**H_E_**								
** D0**	0.821	0.818	0.774	0.631	0.836	0.864	0.820	0.757
** DF**	0.816	0.828	0.856	0.504	0.834	0.867	0.818	0.773

Microsatellite markers and their descriptions are as reported previously [35]. bp- base pairs; D0- day 0; DF- day of failure; No.- number; PCR- polymerase chain reaction; PCR positivity indicates number of samples successfully amplified for the indicated loci. MOI- multiplicity of infection, minimum number of different parasite genotypes present; H_E_-expected virtual heterozygosity where H_E_ = [n/(n –1)] X(1– ∑p^2^i), where n is the number of samples. This variable can be defined as the probability that a randomly chosen pair of alleles differ from each other.

#### Drug level testing

Day 7 whole blood capillary lumefantrine levels from dried blood spots for the patients in the AL arm with recurrent parasitemia ranged from below detection to 1,537.3 ng/mL (median 446.3 ng/mL). Using previously reported cut-off levels identified to predict *P. falciparum* treatment failure of <280 ng/ml [33] and <175 ng/ml [41], 30.4% (14/46) and 19.6% (9/46) of the recurrent samples were below this level, respectively.

Chloroquine and DCQ levels on the day of recurrent parasitemia in the CQ arm ranged from below detection to 485.1 ng/mL. Of those that had recurrent parasitemia by day 28, 30% (3/10) were noted to have CQ+DCQ levels above the minimum effective concentration (i.e. 100 ng/mL). Thus, 2.8% (3/108) of all patients had recurrent parasitemia by day 28 with adequate CQ blood levels, suggesting *P. vivax* CQ resistance. Of the three recurrent parasitemias with adequate CQ concentrations: one was caused by the identical *P. vivax* strain (3-year-old boy) and two were caused by different *P. vivax* strains (7- and 2-year-old boys).

### Adverse Events

Both AL and CQ were well tolerated with no serious adverse events reported. Overall, 48.4% (59/122) of the patients in the AL arm and 50.8% (61/120) in the CQ arm reported at least one adverse event. 8.2% (10/122) of the patients in the AL arm and 12.5% (15/120) in the CQ arm presented with oral ulcers, which was the most common complaint, followed by nausea/vomiting and cough (Table 5).

**Table 5:** Adverse events by treatment arm.

Patients with adverse events, n (%)	Artemether-lumefantrine (N = 122)	Chloroquine (N = 120)
Abdominal Pain	5 (4.1)	9 (7.5)
Anorexia	2 (1.6)	2 (1.7)
Cough	10 (8.2)	7 (5.8)
Diarrhea	6 (4.9)	5 (4.2)
Dizziness	5 (4.1)	1 (0.8)
Headache	4 (3.3)	4 (3.3)
Myalgia	6 (4.9)	2 (1.7)
Nausea/vomiting	9 (7.4)	13 (10.8)
Oral ulcers	10 (8.2)	15 (12.5)
Pruritus	4 (3.3)	7 (5.8)
Rash	4 (3.3)	2 (1.7)

## Discussion

We show that both AL and CQ are well-tolerated and remain efficacious for early *P. vivax* treatment response involving 242 patients from two sites in central Ethiopia in late 2009. In both treatment arms, no recurrent parasitemias were seen prior to 21 days and there were very few patients that remained microscopy positive beyond day 2 in contrast to high rates of day 2 and 3 positivity seen in Asia with known high grade CQ resistance [13], [42]. Albeit low, the CQ arm had higher day 2 positivity compared to the AL arm, which is consistent with conclusions from a recent review of ACTs for *P. vivax* treatment which noted that patients receiving ACTs clear their parasites more quickly (median parasite clearance time = 28.8 hrs) compared to CQ-based monotherapy or non-ACT combination therapies (50.4 hrs) [43].

Although high rates of recurrent parasitemia were noted in both treatment arms, fewer patients receiving CQ presented with recurrent parasitemia by day 28 compared to those receiving AL indicating that CQ may have provided longer post-treatment prophylaxis. Our recurrent *P. vivax* parasitemia findings are higher than some previously reported studies in Ethiopia [24], [25], [44], but are consistent with more recent efficacy studies from 2005 and 2009 [26], [45]. Similarly, observed recurrent parasitemia rates with AL and CQ are comparable to reports from the Greater Mekong Sub-region [16], [17], [46], [47], but not as high as the Southwest Pacific region where rapidly relapsing tropical strains, high grade CQ resistance, and high rates (i.e. 57–70%) of recurrence within 28–42 days following AL administration are common [11], [14], [42]. Even though confirmed CQ resistance appears to be at <5% in our study consistent with others from Ethiopia that included CQ drug level testing [24]–[26], the confirmation of resistant strains, the relatively high recurrent rate seen by day 28 and other reports from elsewhere in Ethiopia of parasitemia recurrences occurring as early as day 7 [45] are all concerning for the possibility of evolving CQ resistance. Continued monitoring involving additional sites is needed to understand the efficacy of antimalarial drugs in Ethiopia, and it is possible that focal drug resistance could emerge within a large and populous country such as Ethiopia.

Our study also highlights issues related to classifying recurrent parasitemia using molecular methods. Although genotyping to distinguish recurrent parasitemias in *P. vivax in vivo* studies is not standardized and is not part of the current standard WHO protocol [30], it has been used to improve characterization of recurrent *P. vivax* parasitemias [16], [18], [48], [49]. The combination of microsatellite markers used in our study reliably differentiated genetic relatedness of parasite populations allowing us to better estimate the true efficacy of *P. vivax* treatment regimens. Recurrent infections with the same genotype (true failure and homologous relapse) were distinguished from those with a different genotype (reinfection or heterologous relapse). Despite the possibility of a recurrent parasitemia arising from a clonal hypnozoite, all identical recurrent parasitemias were classified as treatment failures resulting in a higher, more conservative estimate of treatment failure, but still an improvement over the unadjusted treatment failure rates.

Chloroquine drug level testing on the day of failure as recommended by WHO to identify CQ resistance [30] was helpful in documenting that three out of the ten recurrent parasitemias by day 28 in the CQ arm occurred with CQ+DCQ levels above the minimal effective concentration (i.e. 100 ng/mL of whole blood) [50]. Although, day 7 lumefantrine levels were determined in patients with recurrent parasitemias in the AL arm, the utility of obtaining lumefantrine levels from filter paper to aid in accurately determining treatment failures especially for *P. vivax* remains challenging and was of limited benefit in this study.

Approximately half of the patients in both the AL and CQ reported some adverse events, none were severe and most were consistent with ones generally reported [51], [52], [53], i.e. headaches and nausea/vomiting for AL, and headaches, vomiting, myalgia, and pruritus for CQ. Although we noted oral lesions in both treatment arms, this is not a previously reported common drug side-effect and unlikely to be drug-related.

There are several limitations of this trial including drug administration procedures, baseline differences, and external validity. Due to logistical reasons, the evening doses of AL were not supervised and the antimalarial treatment administration not blinded. The lower day 28 cure rates seen in the AL arm most likely resulted from its shorter post-prophylactic effect, but poor adherence cannot be discounted as a contributing factor, even though, all patients reported having taken their evening doses. Reassuringly, approximately 70% of the patients with recurrent parasitemia had lumefantrine levels >280 ng/mL as compared to only 51% in a study with supervised artemether-benflumetol administration [33]. Although treatment allocation was randomized, the patients in the AL arm were younger than the CQ arm. With the finding that younger age was associated with higher recurrent parasitemia, the contribution of this patient characteristic in the lower AL cure rates by day 28 needs to be considered. Results from this study may not be applicable to other ACTs or other areas with different resistance and relapse patterns. Furthermore, our study was not powered to estimate differences in efficacy across treatment arms or between sites. Finally, the two health facilities were not chosen randomly and thus may not be representative of our target population.

Some experts have suggested unifying malaria treatments under a single ACT regimen [43], [54] in countries where *P. falciparum* and *P. vivax* are sympatric. However, due to a relative short half-life, AL provides less post-exposure prophylaxis against *P. vivax* recurrent parasitemias. In Indonesia, patients receiving DP compared to AL were half as likely to be anemic and 6.6 times less likely to carry *P. vivax* gametocytes [53]. In a setting where primaquine is not routinely administered (e.g. Ethiopia), CQ resulted in fewer early recurrent parasitemias than AL, which could provide some hematologic and transmission benefits. This supports the maintenance of current species-specific treatment policy for Ethiopia [9].

With high recurrent parasitemia rates for both AL and CQ at 42 days following treatment, the addition of primaquine for radical cure should be considered in all areas of Ethiopia as is currently recommended by WHO [27]. Furthermore, with an increasing number of reports showing that *P. vivax* infections can lead to severe disease [55], [56], [57], country policies and practices that fail to prevent substantial numbers of relapsing *P. vivax* illnesses need to be reexamined. In Ethiopia, the addition of primaquine to CQ decreased the cumulative incidence of therapeutic failure at day 28 by a life-table analysis method from 5.76% to 0.75% and the cumulative risk of relapse at day 157 by a life-table method from 61.8% to 26.3% [44]. Along with continued surveillance of AL or CQ drug resistance, the added benefit of co-administering primaquine with AL or CQ should be explored urgently in Ethiopia.

## Supporting Information

Table S1 Microsatellite diversity for the eight markers for all samples with recurrent parasitemia at both day 0 (pre-treatment) and day of failure.
(DOCX)

Checklist S1 **CONSORT checklist.**
(DOC)

Protocol S1 **Study protocol.**
(DOC)
