# Supplementary material for: In Vivo Efficacy of Artemether-Lumefantrine and Chloroquine against Plasmodium vivax: A Randomized Open Label Trial in Central Ethiopia
Source: PLoS One. 2013 May 22;8(5):e63433. doi: 10.1371/journal.pone.0063433 (PMC3661577; doi:10.1371/journal.pone.0063433)
Supplement: Table S1 — Microsatellite diversity for the eight markers for all samples with recurrent parasitemia at both day 0 (pre-treatment) and day of failure. (DOCX) [file pone.0063433.s001.docx]

**Table S1. Microsatellite diversity for the eight markers for all samples with recurrent parasitemia at both day 0 (pre-treatment) and day of failure**

|  |  |  | Microsatellites | | | | | | | |
| --- | --- | --- | --- | --- | --- | --- | --- | --- | --- | --- |
| Sample | Number | Day | v23 | v24 | v25 | v27 | v29 | v30 | v32 | v33 |
| 2018 | 1 | 0 | 273 | 162 | X | X | 246 | 151 | 116 | 117 |
|  |  | DF | 273 | 162 | X | X | 246 | 152 | 126 | 117 |
| 2020 | 2 | 0 | 272 | 162 | 100, 114 | 159 | 231 | 145, 155 | 116 | 117, 121 |
|  |  | DF | 272 | 190 | 115 | X | 250 | 156 | 118 | 117 |
| 2027 | 3 | 0 | 270 | 164 | 100 | 159 | 252 | 152 | 116 | 117 |
|  |  | DF | 271 | 162 | 100 | 167 | 243 | 151 | 126 | 117 |
| 2133 | 4 | 0 | 269 | 161 | 119 | X | 246 | 151 | 116 | 117 |
|  |  | DF | 272 | 196 | 119 | X | 246 | 152 | 116 | 117 |
| 2139 | 5 | 0 | 269 | 162 | X | X | 252 | 151 | 116 | 117 |
|  |  | DF | 269 | 162 | X | X | 252 | 149 | 116 | 117 |
| 3003 | 6 | 0 | 269 | 160 | 107 | 167 | 231, 240 | 155 | 118 | 117 |
|  |  | DF | 269 | 184 | 107 | 167 | 231, 239 | 153 | 118 | 117 |
| 3007 | 7 | 0 | 271 | 196 | 103, 117 | 159 | 244 | 153 | 122 | 119 |
|  |  | DF | 271 | 196 | 103, 117 | 159 | 244 | 155 | 122 | 119 |
| 3009 | 8 | 0 | 271 | 156 | 100, 110 | 159 | 231 | 153 | 116 | 120, 128 |
|  |  | DF | 270 | 164 | 105 | X | 243 | 155 | 116 | 117 |
| 3010 | 9 | 0 | 269 | 196 | X | X | 246 | 147 | 116 | 121 |
|  |  | DF | 269 | 196 | X | X | 246 | 146 | 116 | 121 |
| 3017 | 10 | 0 | 273 | 166 | 101 | 160 | 244 | 151 | 116 | 117 |
|  |  | DF | 272 | 166 | 100 | 159 | 244 | 151 | 116 | 117 |
| 3019 | 11 | 0 | 268 | 162 | 100, 116 | 167 | 244 | 143 | 118 | 117 |
|  |  | DF | 269 | 163 | 101, 116 | X | 232 | 142 | 118 | 116 |
| 3025 | 12 | 0 | 273 | 170 | X | X | 244 | 149 | 122 | 121 |
|  |  | DF | 272 | 170 | X | X | 244 | 152 | 121 | 121 |
| 3034 | 13 | 0 | 269 | 160 | 100 | 160 | 251 | 151 | 116 | 116 |
|  |  | DF | 271 | 160, 170 | 100, 114 | 167 | 246 | 151 | 122 | 127 |
| 3038 | 14 | 0 | 273 | 166 | 100 | X | 240 | 152 | 126 | 128 |
|  |  | DF | 273 | 166 | 100 | X | 240 | 152 | 126 | 128 |
| 3047 | 15 | 0 | 269 | X | X | X | 246 | 147 | 116 | 120 |
|  |  | DF | 269 | 162 | X | X | 246 | 147 | 116 | 121 |
| 3054 | 16 | 0 | 271 | 160 | 101 | X | 252 | 147 | 126 | 127 |
|  |  | DF | 270 | 160 | 100 | X | X | 151 | 126 | 126 |
| 3056 | 17 | 0 | 273 | 162 | 100 | X | 241 | 156 | 126 | 121 |
|  |  | DF | 273 | 162 | 100 | X | 242 | 151 | 126 | 121 |
| 3059 | 18 | 0 | 273 | 166 | 100 | 159 | 240 | 151 | 126 | 128 |
|  |  | DF | 272 | 167 | 100 | X | 240 | 152 | 126 | 128 |
| 3060 | 19 | 0 | 269 | 164 | 100 | X | 244 | 153 | 116 | 121 |
|  |  | DF | 269 | 164 | 100 | X | 244 | 153 | 116 | 121 |
| 3062 | 20 | 0 | 270 | 160 | 101 | 159 | 245 | 150 | 118 | 125 |
|  |  | DF | 271 | 160 | 101 | X | 243 | 150 | 118 | 125 |
| 3065 | 21 | 0 | 270 | 164 | 100 | 159 | 246 | 152 | 118 | 113 |
|  |  | DF | 273 | 162 | 100 | 159 | 246 | 151 | 118 | 128 |
| 3066 | 22 | 0 | 263 | 164 | 100 | X | 240 | 151 | 116 | 116 |
|  |  | DF | 263 | 164 | 100 | X | 241 | 153 | 116 | 115 |
| 3069 | 23 | 0 | 272 | 166 | X | X | 245 | 151 | 118 | 117 |
|  |  | DF | 273 | 166 | 100 | X | 244 | 156 | 118 | 115 |
| 3072 | 24 | 0 | 271 | 162 | 100, 114 | 166 | 244 | 151 | 116 | 117, 127 |
|  |  | DF | 269 | 162 | 100, 114 | 159 | 244 | 151 | 126 | 127 |
| 3077 | 25 | 0 | 273 | 160 | 105 | X | 244 | 136 | 118 | 121 |
|  |  | DF | 272 | 160 | 103, 117 | X | 250 | 135 | 120 | 121 |
| 3079 | 26 | 0 | 271 | 160 | 100 | 158 | 244 | 151 | 116 | 117 |
|  |  | DF | 271 | 160 | 105 | X | 244 | 151 | 116 | 117 |
| 3080 | 27 | 0 | 271 | 160 | 113 | 158 | 244 | 155 | 124 | 117 |
|  |  | DF | 271 | 164 | 115 | X | 244 | 159 | 122 | 117, 127 |
| 3081 | 28 | 0 | 270 | 160 | 113 | 159 | 244 | 153 | 123 | 117 |
|  |  | DF | 271 | 164 | 103 | X | 244 | 155 | 122 | 117 |
| 3106 | 29 | 0 | 273 | 162 | 104 | X | 246 | 155 | 122 | 117 |
|  |  | DF | 272 | 162 | 100 | X | 246 | 155 | 122 | 117 |
| 3116 | 30 | 0 | 269 | 162 | 100, 112 | X | 245 | 151 | 122 | 117 |
|  |  | DF | 269 | 162 | 101, 112 | X | 239 | 151 | 122 | 117 |
| 3123 | 31 | 0 | 263 | 162 | 115 | 169 | 244 | 136, 151 | 116 | 119 |
|  |  | DF | 271 | 162 | 103, 117 | X | 246 | 155 | 121 | 117 |
| 3124 | 32 | 0 | 269 | 162 | 100 | X | 245 | 153 | 130 | 117 |
|  |  | DF | 269 | 162 | 107 | X | 244 | 155 | 130 | 117 |
| 3125 | 33 | 0 | 269 | 166 | 100 | X | 244 | 142 | 126 | 117, 127 |
|  |  | DF | 269 | 166 | X | 159 | 243 | 142 | 126 | 116 |
| 3127 | 34 | 0 | 271 | 156 | 105 | 167 | 246 | 151 | 126 | 117 |
|  |  | DF | 270 | 156 | 103 | 167 | 244 | 155 | 126 | 117 |
| 3128 | 35 | 0 | 272 | 160 | 104 | 159 | 231, 246 | 151 | 124 | 117,128 |
|  |  | DF | 272 | 160 | 104 | 159 | 231, 246 | 152 | 126 | 117 |
| 3132 | 36 | 0 | 269 | 176 | 100 | X | 244 | 148 | 116 | 127 |
|  |  | DF | 269 | 176 | 100 | X | 244 | 150 | 116 | 127 |
| 3135 | 37 | 0 | 271 | 184 | 100 | 159 | 231 | 154 | 118 | 125 |
|  |  | DF | 271 | 184 | 100 | 159 | 231 | 154 | 118 | 125 |
| 3138 | 38 | 0 | 271 | 164 | 100 | 159 | 246 | 152 | 118 | 112 |
|  |  | DF | 272 | 162 | 109 | X | 246 | 152 | 120 | 128 |
| 3142 | 39 | 0 | 271 | 162 | 100 | X | 250 | 155 | 118 | 120 |
|  |  | DF | 271 | 162 | X | X | 244 | 173 | 118 | 121 |
| 3145 | 40 | 0 | 272 | 162 | 104 | X | 246 | 173 | 116 | 116 |
|  |  | DF | 273 | 162 | 101 | X | 246 | 173 | 116 | 117 |
| 3146 | 41 | 0 | 272 | 166 | 100, 112 | 167 | 244 | 151 | 116 | 116 |
|  |  | DF | 273 | 162 | X | 167 | 244 | 155 | 122 | 121 |
| 3148 | 42 | 0 | 272 | 162 | 100 | 167 | 231 | 151 | 118 | 121 |
|  |  | DF | 273 | 162 | 100 | 167 | 231 | 155 | 118 | 121 |
| 3150 | 43 | 0 | 270 | 184 | 100 | 159 | 231 | 149 | 118 | 125 |
|  |  | DF | 270 | 184 | 100 | X | 231 | 149 | 118 | 125 |
| 3152 | 44 | 0 | 271 | 168 | 100 | X | 244 | 153 | 118 | 116 |
|  |  | DF | 271 | 168 | 101 | X | 243 | 153 | 118 | 117 |
| 3153 | 45 | 0 | 273 | 162 | 100 | 159 | 244 | 153 | 118 | 128 |
|  |  | DF | 272 | 162 | 100 | 159 | 244 | 153 | 118 | 128 |
| 3159 | 46 | 0 | 272 | 162 | 117 | X | 244 | 152 | 122 | 124 |
|  |  | DF | 271 | 161 | 117 | 167 | 246 | 151 | 118 | 125 |
| 3161 | 47 | 0 | 272 | 166 | 100 | X | 240 | 147 | 126 | 128 |
|  |  | DF | 272 | 166 | 100 | X | 239 | 153 | 126 | 128 |
| 3162 | 48 | 0 | 273 | 162 | 100 | 159 | 244 | 155 | 118 | 117 |
|  |  | DF | 272 | 160 | 101 | X | 244 | 155 | 120 | 120 |
| 3165 | 49 | 0 | 271 | 156 | 101 | X | 241 | 147 | 126 | 117 |
|  |  | DF | 272 | 160 | 117 | 159 | 243 | 151 | 118 | 117 |
| 3169 | 50 | 0 | 267 | 162 | X | 159 | 252 | 151 | 126 | 127 |
|  |  | DF | 269 | 162 | X | X | 252 | 152 | 126 | 127 |
| 3170 | 51 | 0 | 272 | 166 | 100 | X | 244 | 153 | 118 | 117 |
|  |  | DF | 273 | 166 | 117 | 159 | 244 | 155 | 118 | 117 |
| 3172 | 52 | 0 | 271 | 184 | 100 | 159 | 231 | 154 | 118 | 125 |
|  |  | DF | 271 | 184 | 100 | 159 | 231 | 153 | 118 | 125 |
| 3173 | 53 | 0 | 272 | 160 | 100, 118 | X | 244 | 149 | 122 | 117 |
|  |  | DF | 272 | 160 | 104, 118 | X | 246 | 151 | 122 | 117 |
| 3176 | 54 | 0 | 273 | 160 | 117 | 169 | 250 | 143 | 128 | 117 |
|  |  | DF | 272 | 160 | 115 | 169 | 250 | 143 | 116 | 117 |
| 3179 | 55 | 0 | 271 | 162 | 107 | X | 244 | 150 | 130 | 113, 128 |
|  |  | DF | 271 | 162 | X | X | 244 | 145, 150 | 118 | 113 |
| 3183 | 56 | 0 | 269 | 156 | 100, 114 | X | 246 | 155, 169 | 118 | 117 |
|  |  | DF | 272 | 162 | 100 | X | 231, 246 | 150 | 118 | 117 |
| 3184 | 57 | 0 | 271 | 156 | X | 159 | 246 | 150 | 116 | 117 |
|  |  | DF | 271 | 155 | X | 159 | 246 | 150 | 116 | 117 |
| 3185 | 58 | 0 | 263 | 166 | 115 | 159 | 231 | 151 | 118 | 128 |
|  |  | DF | 263 | 166 | 115 | 159 | 231 | 151 | 118 | 128 |
| 3188 | 59 | 0 | 263 | 162 | 100 | X | 248 | 151 | 116 | 117, 127 |
|  |  | DF | 263 | 162 | 100 | X | 248 | 151 | 116 | 117 |
| 3189 | 60 | 0 | 273 | 160 | 113 | 167 | 240 | 153 | 120 | 117, 121 |
|  |  | DF | 271 | 160 | 113 | X | 244 | 155 | 126 | 117 |
| 3192 | 61 | 0 | 269 | X | 103 | 158 | X | 154 | 116 | 121 |
|  |  | DF | 269 | X | 103 | X | 252 | 153 | 116 | 121 |
| 3193 | 62 | 0 | 271 | 162 | 117 | 169 | 246 | 151 | 118 | 117 |
|  |  | DF | 263, 271 | 162 | 117 | 169 | 244 | 153 | 118 | 116 |
| 3194 | 63 | 0 | 272 | 160 | X | X | 248 | 156 | 117 | 117 |
|  |  | DF | 272 | 160 | X | X | 248 | 155 | 128 | 117 |
| 3196 | 64 | 0 | 263 | 162 | 100 | X | 252 | 151 | 117 | 117 |
|  |  | DF | 263 | 162 | 100 | X | 231 | 151 | 122 | 116 |
| 3197 | 65 | 0 | 272 | 160 | 100, 118 | X | 216, 243 | 151 | 122 | 116 |
|  |  | DF | 272 | X | 100, 118 | X | 244 | 151 | 122 | 116 |
| 3201 | 66 | 0 | 271 | 156, 162, 166 | 100 | 159 | 231 | 151 | 122 | 113 |
|  |  | DF | 271 | 166 | 100 | X | 244 | 151 | 118 | 113 |
| 3202 | 67 | 0 | 271 | 162 | 104 | X | X | 151 | 126 | 117 |
|  |  | DF | 273 | 184 | 103 | X | 232 | 155 | 118 | 125 |
| 3205 | 68 | 0 | 273 | 156, 166 | 100 | X | 244 | 156 | 122 | 116 |
|  |  | DF | 273 | 156, 166 | X | X | 244 | 155 | 122 | 117 |
| 3206 | 69 | 0 | 269 | 196 | 117 | X | 246 | 147 | 116 | 121 |
|  |  | DF | 269 | 196 | X | X | 246 | 146 | 116 | 121 |
| 3207 | 70 | 0 | 271 | 184 | 100 | 159 | 231 | 153 | 118 | 125 |
|  |  | DF | 270 | 156 | 103 | X | 231 | 149 | 118 | 125 |
| 3214 | 71 | 0 | 270 | 184 | 100 | X | 231 | 154 | 118 | 124 |
|  |  | DF | 271 | 184 | 100 | X | 231 | 153 | 118 | 125 |
| 3216 | 72 | 0 | 263 | 162 | 100, 112 | 159 | 244 | 155 | 126 | 117 |
|  |  | DF | 263 | 164 | 100, 112 | X | 245 | 155 | 126 | 116 |
| 3219 | 73 | 0 | 272 | 160 | 106 | 159 | 250 | 136 | 122 | 116 |
|  |  | DF | 272 | 160 | 106 | 159 | 250 | 136 | 115 | 116 |
| 3223 | 74 | 0 | 272 | 162 | 100 | X | 244 | 152 | 126 | 112 |
|  |  | DF | 272 | 162 | 111 | X | 246 | 151 | 126 | 112 |
| 3225 | 75 | 0 | 271 | 170 | 100, 114 | X | 252 | 156 | 120 | 117 |
|  |  | DF | 271 | X | 100 | X | 252 | 155 | 134 | 119 |
| 3227 | 76 | 0 | 271 | 196 | 103 | X | 248 | 143 | 128 | 127 |
|  |  | DF | 270 | 196 | 103 | X | 248 | 143 | 128 | 127 |
| 3230 | 77 | 0 | 271 | 160 | 109 | 159 | 244 | 150 | 117 | 117 |
|  |  | DF | 271 | 160 | 109 | 159 | 244 | 150 | X | 117 |
| 3233 | 78 | 0 | 273 | 162 | 125 | 159 | 250 | 136 | 118 | 121 |
|  |  | DF | 283 | 160 | 113, 125 | 166 | 250 | 145 | 122 | 117 |
| 3234 | 79 | 0 | 271 | 162 | 100 | X | 246 | X | 116 | 117 |
|  |  | DF | 271 | 162 | 103 | X | 246 | 169 | 116 | 117 |
| 3235 | 80 | 0 | 273 | 156 | 113 | X | 250 | 136 | 120 | 121 |
|  |  | DF | 273 | 196 | 107 | X | 246 | 145 | 126 | 123 |
| 3237 | 81 | 0 | 263 | 161 | 100 | X | 231 | 152 | 117 | 116 |
|  |  | DF | 263 | 162 | 100 | X | 231 | 151 | 122 | 117 |

Abbreviations: DF- Day of Failure; X=not detected

Shaded sample pairs classified as identical genotypes. Unshaded sample pairs classified as different genotypes with greater than 2 basepair difference detected in at least one out of 8 markers between the Day 0 (pretreatment) and day of failure
