# Supplementary material for: In Vivo Efficacy of Artemether-Lumefantrine and Chloroquine against Plasmodium vivax: A Randomized Open Label Trial in Central Ethiopia
Source: PLoS One. 2013 May 22;8(5):e63433. doi: 10.1371/journal.pone.0063433 (PMC3661577; doi:10.1371/journal.pone.0063433)
Supplement: Protocol S1 — Study protocol. (DOC) [file pone.0063433.s003.doc]

**TRIAL PROTOCOL**

**Ethiopia *in-vivo* efficacy study 2009:**

**Evaluating the efficacy of artemether-lumefantrine for the treatment of uncomplicated *Plasmodium falciparum* infection and either artemether-lumefantrine or chloroquine for *P. vivax* infection**

**COLLABORATORS**

**Centers for Disease Control and Prevention (CDC):**

Jimee Hwang, MD MPH (ethics number 1008) will be responsible for protocol development, data management and analysis, and preparation of reports

Scott Filler, MD DTM&H (ethics number 2642) will supervise protocol development and preparation of reports.

Hoang Dang MPH (ethics number 3030) will provide logistic support.

S. Patrick Kachur, MD MPH (ethics number 6519) will supervise protocol development and preparation of reports.

Laurence Slutsker MD MPH (ethics number 15856) will supervise protocol development and preparation of reports.

Venkatachalam Udhayakumar PhD (ethics number 13427) will provide oversight of molecular laboratory work at CDC.

Tauqeer Alam PhD (ethics number 7336) will assist in the molecular laboratory work at CDC.

**Ethiopia Health and Nutrition Research Institute (EHNRI):**

Moges Kassa, MSc will provide oversight of molecular laboratory work at EHNRI.

**Federal Ministry of Health (FMOH):**

Daddi Jima, MD, MPH will review protocol development and preparation of reports.

**International Center for AIDS Care and Treatment Programs (ICAP):**

Zenebe Melaku, MD will supervise protocol development and the implementation of the study.

Bereket HaileGiorgis, MD MSc will assist in protocol development, be responsible for the implementation of the study, and the training and supervision of staff members.

Samuel Girma, MD will assist in the implementation of the study and the training and supervision of staff members.

David Hoos, MD MPH will supervise protocol development and the implementation of the study.

**Oromia Regional Health Bureau:**

Kedir Gobena, BSC will facilitate logistical support for the project at the regional level.

**United States Agency for International Development (USAID):**

Richard Reithinger, PhD will review protocol development and preparation of reports.

Dereje Olana, BSC MSc will review protocol development and preparation of reports.

**Expected Dates: August 2009- August 2010**

**Location: Oromia Regional State, Ethiopia
Sites: 1) Adama Malaria Training Center, 2) Awash Melkasa Health Center, and 3) Debrezeit Malaria Laboratory**

Table of Contents

[SUMMARY 3](#__RefHeading___Toc231788309)

[INTRODUCTION 4](#__RefHeading___Toc231788310)

[OBJECTIVES 6](#__RefHeading___Toc231788311)

[STUDY DESIGN 7](#__RefHeading___Toc231788312)

[Study Sites 7](#__RefHeading___Toc231788313)

[Drugs 7](#__RefHeading___Toc231788314)

[Population 9](#__RefHeading___Toc231788315)

[SCREENING AND ENROLLMENT PROCEDURES 11](#__RefHeading___Toc231788316)

[FOLLOW-UP PROCEDURES 13](#__RefHeading___Toc231788317)

[END-POINTS 13](#__RefHeading___Toc231788318)

[DATA HANDLING AND ANALYSIS 15](#__RefHeading___Toc231788319)

[HANDLING OF UNEXPECTED OR ADVERSE EVENTS 17](#__RefHeading___Toc231788320)

[ETHICAL ISSUES 19](#__RefHeading___Toc231788321)

[DISSEMINATION, NOTIFICATION AND REPORTING OF RESULTS 22](#__RefHeading___Toc231788322)

[REFERENCES 24](#__RefHeading___Toc231788323)

[ANNEX 26](#__RefHeading___Toc231788324)

[ANNEX I *P. falciparum* treatment outcomes 26](#__RefHeading___Toc231788325)

[ANNEX II *P. vivax* treatment outcomes 27](#__RefHeading___Toc231788326)

[ANNEX III Severe malaria definitions 28](#__RefHeading___Toc231788327)

[ANNEX IV Weight for Height Chart 29](#__RefHeading___Toc231788411)

[ANNEX V Schedule of follow-up activities 30](#__RefHeading___Toc231788412)

[ANNEX VI Study Schedule 31](#__RefHeading___Toc231788413)

[ANNEX VII Consent/Assent Form 32](#__RefHeading___Toc231788414)

[ANNEX VIII Data Safety Monitoring Board 40](#__RefHeading___Toc231788418)

# SUMMARY

Following the rapid development of significant drug resistance of *Plasmodium falciparum* (Pf) to chloroquine and then sulfadoxine-pyrimethamine (the first line therapy in Ethiopia 1998-2004), artemether- lumefantrine (Coartem or AL) was adopted as first line therapy in Ethiopia in 2004. According to the current national malaria diagnosis and treatment guidelines, first-line treatment for uncomplicated falciparum infection is AL. First-line treatment for *Plasmodium vivax* (Pv) is with chloroquine (CQ) alone without primaquine therapy in malarious areas. For all clinical infection without laboratory confirmation, AL which is effective against both Pf and Pv is the first-line treatment. Thus, in Ethiopia, where treatment for malaria without laboratory confirmation occurs frequently, Pv is often treated with AL as the standard of care. Furthermore, World Health Organization (WHO) recommends AL for the treatment of Pv, where AL has been adopted as first-line treatment for Pf. Now with wide-spread use of AL and CQ, we propose to conduct an antimalarial efficacy study to monitor the effectiveness of these therapies in Ethiopia and to determine how efficacious these drugs remain. This information will inform future policy changes with respect to appropriate antimalarial strategies.

The simplest and most universally accepted measure of testing for antimalarial drug treatment efficacy, the standardized procedures outlined in the World Health Organization *Assessment and monitoring of antimalarial drug efficacy for the treatment of uncomplicated falciparum malaria* and the WHO *Monitoring antimalarial drug resistance*, will be followed.

In this proposal, patients aged above 6 months with symptomatic malaria presenting to health centers will be enrolled for treatment with AL for *P. falciparum* infection, and either AL or CQ for *P. vivax* infection. Clinical, parasitologic, and hematologic parameters will be monitored for *P. falciparum* and *P. vivax* infection over a 42-day follow-up period, which will be used to evaluate drug efficacy. Results from this research study will be used to assist Ethiopia in assessing their current national malaria drug policies.

# INTRODUCTION

The impact of malaria on the health and economic development of human populations is greatest in the tropics and sub-tropics [1]. The World Health Organization (WHO) has estimated 247 million cases of malaria and 881 000 deaths occurred in 2006 making it one of the world's leading killers [2]. Children under 5 years of age living in sub-Saharan Africa account for the majority of the illness and death caused by malaria worldwide. There are substantially less data on individuals 5 years of age and above.

Most countries in sub-Saharan Africa have adopted the WHO Global Strategy for Malaria Control, which relies primarily on prompt and effective antimalarial treatment. The ultimate success of this strategy rests on the ability of governments to provide antimalarial drugs which are indeed efficacious. Because of wide-spread and intensifying antimalarial drug resistance to a variety of currently available antimalarial drugs, the decision of which drug to recommend becomes both critical and complex.

Drug efficacy assessment methodology appropriate to the situation existing in sub-Saharan Africa has been developed over the last 20 to 25 years. The goals of these studies are to assess antimalarials currently being used for the treatment of uncomplicated malaria. These data are critical for guiding the development of rational antimalarial drug policies and treatment guidelines. The duration of the resolution of symptoms, the proportion of patients experiencing a “clinical failure” within a pre-determined follow-up period, and the ability of anemic patients to improve their hematologic status have become the primary indicators of antimalarial drug efficacy [3].

This document presents a standardized protocol for assessing the therapeutic efficacy of antimalarial drugs in Africa utilizing the most current concepts of not only what data are needed to accurately judge antimalarial efficacy, but also what information is required by national decision makers to devise rational policies and guidelines for their countries. This protocol is based on experiences gained in drug efficacy testing in numerous countries throughout the world and follows a standardized WHO protocol for drug efficacy testing [4, 5].

Malaria in Ethiopia is highly unstable, with potential for epidemics. In Ethiopia, malaria transmission is largely determined by climate and altitude. Most of the transmission occurs between September and December, after the main rainy season from June to August. Certain areas, largely in the eastern part of the country including parts of Oromia, experience a second minor transmission period from April to May, following a short rainy season from February to March. Five main malaria eco-epidemiological strata are recognized:

- Stable, year round, transmission in the western lowlands and river basin areas of Gambella;
- Seasonal transmission in lowland areas <1,500 meters above sea level (m);
- Epidemic-prone areas in highland fringes between 1,500 – 2,500 m;
- Arid areas where malaria is only found near semi-permanent water bodies; and
- Malaria-free highland areas >2,500 meters.

Malaria is the leading communicable disease in Ethiopia. Overall, malaria accounts for up to 17% of outpatient consultations, 15% of admissions, and 29% of in-patient deaths (Federal Ministry of Health data, 2005/6). About 75% of the country is malarious (defined as *areas <2000 m*), with about 68% (i.e. 52 million) of the country’s total population living in areas at risk of malaria. Approximately 9.5 million clinical cases of malaria were reported annually between 2001– 2005 (range: 8.4 – 11.5 million), with an annual average of 487,984 laboratory confirmed cases over the same period (range: 392,419 – 591,442) (Federal Ministry of Health data, 2005/6). Approximately 70,000 people die of malaria each year in Ethiopia (Federal Ministry of Health data, 2005/6). Similarly, in Oromia Regional State malaria is considered to be the most important communicable disease. Three quarters of the region, i.e. 262 of 297 (88%) districts and 4,237 of 6,765 (63%) communities, are considered malarious, accounting for over 17 million persons at risk of infection. There are an estimated 1.5 to 2 million clinical cases per year, with malaria accounting for 20-35% of outpatient consultations, 16% of hospital admissions, and 18-30% of hospital deaths in the region.

*P. falciparum* (Pf) and *P. vivax* (Pv) are the two dominant parasite species with relative frequencies of 60% and 40%, respectively, although this proportion varies in geography and time with published ranges of 41-89% for Pf and 11-55% for Pv [6, 7]. Chloroquine (CQ) resistant Pf was first reported from Africa in 1978 [8] and became a major public health threat to Ethiopia in the 1990s. An evaluation conducted in Ethiopia from 1989–1991 noted overall 3.7% of those treated with chloroquine for falciparum malaria returned to the clinic within 2 weeks due to not being cured but 87.2% of those who returned demonstrated chloroquine resistance [9]. Reports of unsuccessful treatment of *P. falciparum* with chloroquine increased with time. By the late 1990s, 86-88% treatment failure of Pf to chloroquine were being reported which prompted the change of first-line treatment of Pf to sulphadoxine-pyrimethamine (SP) in 1998 [10, 11]. Studies evaluating Pf resistance to SP in 2001–2002 showed 2.6-32.0% treatment failure throughout Ethiopia [12-14]. Only a year later in 2003, a nation-wide study involving 11 sentinel sites showed 35.9% and 71.7% treatment failure with 14-day and 28-day follow-up, respectively. Falciparum malaria treatment failure of AL was noted to be 0.9% at baseline prior to its introduction in Ethiopia in 2004 [15]. Recent AL efficacy study for Pf by Aklilu Lemma Institute of Pathobiology in Alaba Health Center, Southern Nations, Nationalities, and People's Region (SNNPR), shows 0% resistance (paper in press). PCR corrected cure rates from other African countries has been also shown to be high (93.9–99%) [16-19].

Chloroquine is the first-line treatment for Pv malaria in Ethiopia, but with the spread of CQ-resistant Pf, Ethiopia recommends AL for the treatment of confirmed Pf and all cases of clinical malaria where diagnostics to determine the specific malaria species are not available. In areas co-endemic for Pf and Pv malaria, such as in Ethiopia, the adoption of AL as first-line Pf treatment raises the inevitability of a significant proportion of Pv malaria being treated with AL. Therefore, World Health Organization (WHO) recommends AL for the treatment of Pv, where AL has been adopted as first-line treatment for Pf. They also state that Pv response to ACT treatment is as good as or better than in falciparum malaria [20]. A study in Thailand demonstrated that the combination of AL and primaquine was as effective and well tolerated compared to chloroquine and primaquine for the treatment of Pv [21], whereas, an earlier study comparing CQ to artemether alone demonstrated no early treatment failures in either arm [22]. This proposed study would provide information on the response to AL treatment for Pv in Ethiopia where falciparum and vivax malaria coexist. Chloroquine resistant Pv has remained rare in Africa with published resistance level of 2-4.6% in Ethiopia [11, 23]. In the recent past we have seen rapid development of resistance to newly introduced anti-malarial therapies, for this reason, it is critical to continuously monitor drug efficacy, to allow sufficient time to change national policy when drug efficacy begins to decline.

*P. vivax* unlike *P. falciparum* has a dormant stage [hypnozoites] that can persist in the liver and cause relapses by invading the bloodstream weeks, or even years later. Both CQ and AL treat only the blood stage parasites and have no impact on the liver stage infection. Primaquine can be used to eliminate hypnozoite forms (radical cure) after the acute infection (clinical cure) has been treated with either CQ or AL. This is often done in malaria-free areas such as the United States. However, it is not routinely done in malaria endemic areas. The national treatment guidelines in Ethiopia state that ”in malarious areas where there is a high risk of reinfection, and where the main purpose of treatment should be to bring about clinical cure rather than radical cure, administration of primaquine is not recommended [24].” In complying with national guidelines, our study will not administer primaquine to attempt radical cure but seek to evaluate the efficacy of CQ and AL in the immediate infection only.

# OBJECTIVES

To assess the therapeutic efficacy of AL for uncomplicated *P. falciparum* and *P. vivax* infections and chloroquine for *P. vivax* infection based on parasitologic, clinical, and hematologic parameters, we will conduct an open-label one-arm in-vivo efficacy assessment for *P. falciparum* and two-arm for *P. vivax*.

**Specific Objectives**

1. To measure the clinical and parasitological efficacy of current first-line therapies of AL for uncomplicated *P. falciparum*, by determining the proportion of patients with Early Treatment Failures (ETF), Late Clinical Failures (LCF), Late Parasitological Failures (LPF), or with Adequate Clinical and Parasitological Response (ACPR) during a 42-day follow-up period for *P. falciparum*, (See Annex I for WHO definitions of treatment outcomes for falciparum malaria)
2. To measure the treatment failure of AL and CQ for *P. vivax* infection during a 42-day follow-up period. (See Annex II for WHO definitions of *P. vivax* treatment failures)
3. To differentiate treatment failures with molecular testing and drug levels

# STUDY DESIGN

### Study Sites

Three potential evaluation sites near to Adama town in Oromia Regional State were selected based on availability of adequate numbers of cases of symptomatic, uncomplicated *P. falciparum* and *P. vivax* malaria infections, willingness of the selected health care facility staff to participate in the study and to support the work with laboratory space, and its proximity to Adama Reference Laboratory (Malaria Training Center). Adama, better known as Nazreth, is a [city](http://en.wikipedia.org/wiki/City) in central [Ethiopia](http://en.wikipedia.org/wiki/Ethiopia) and is located in the [East Shewa Zone](http://en.wikipedia.org/wiki/Misraq_Shewa_Zone) of Oromia, at an elevation of 1712 meters, approximately 100 [km](http://en.wikipedia.org/wiki/Kilometre) southeast of [Addis Ababa](http://en.wikipedia.org/wiki/Addis_Ababa). Based on figures from the [Central Statistical Agency](http://en.wikipedia.org/wiki/Central_Statistical_Agency_(Ethiopia)) in 2005, this city has an estimated total population of 228,623.

The three potential health care facilities selected are:

1. Adama Malaria Training Center located in Adama town
2. Awash Melkasa Health Center (HC) located in Awash Melkasa town about 10 km from Adama town
3. Debrezeit Malaria Laboratory located approximately 50 km northwest of Adama Town.

Data from the three health care facilities collected in the past two months show a range of 7-42 cases of microscopy confirmed Pf and 14-399 cases of microscopy confirmed Pv. We would expect the number of malaria cases and especially Pf cases to increase during the high transmission season (October-December).

| Site | Oct-Dec 2006 | | Oct-Dec 2007 | | July-Aug 2008 | |
| --- | --- | --- | --- | --- | --- | --- |
|  | Pf | Pv | Pf | Pv | Pf | Pv |
| Adama Malaria Training Center |  |  | 37 | 158 | 20 | 91 |
| Awash Melkassa Health Center | 171 | 163 |  |  | 42 | 14 |
| Debrezeit Malaria Center | 941 | 1727 |  |  | 7 | 399 |

However, in Oromia state as well as all of Ethiopia, malaria incidence has been decreasing rapidly due to scale-up of preventive measures e.g. mass distribution of long-lasting insecticidal nets and indoor residual spraying. We will be tracking the number of malaria cases presenting to the sites in the weeks leading up to the study initiation. If the numbers are inadequate at the three suggested sites, we will consider adding or switching sites to ensure enrollment of sufficient cases in a timely manner. All potential health centers are under the jurisdiction of the Oromia Regional Health Bureau and the Ministry of Health. If the numbers are inadequate at the 3 selected sites and 1 alternate site previously named, we will select additional health care centers operated by the Oromia Regional Health Bureau in Oromia State to participate in this study based on the same criteria: location in Oromia Regional State, availability of adequate number of cases of symptomatic, uncomplicated *P. falciparum* and *P. vivax* malaria infections, willingness of the selected health care facility staff to participate in the study and to support the work with laboratory space, and proximity to Adama Reference Laboratory.

### Drugs

**Known efficacy and adverse effects of treatment drugs**

**● Artemether - lumefantrine (Coartem or AL):** Artemether-lumefantrine is a WHO prequalified anti-malarial; WHO certifies the quality, safety and efficacy of the medication, and verifies that the manufacturer complies with WHO Good Manufacturing Practices. AL is currently the first line anti-malarial recommended by Ethiopian Ministry of Health for the treatment of uncomplicated falciparum infection and treatment of clinical malaria cases without laboratory confirmation which comprises of non-malaria cases, falciparum cases, and vivax cases. Adverse events are generally mild, most commonly GI (vomiting and diarrhea) and hematologic (anemia and eosinophilia) [16]. Its safety was demonstrated in studies from Ethiopia and Uganda [15, 25].

- **Chloroquine (CQ):** Chloroquine, a 4-aminoquinoline, was recognized as an effective and safe antimalarial in 1946, and was the most widely used drug against malaria until significant resistance to chloroquine rendered it ineffective against Pf. CQ is currently recommended by Ethiopian Ministry of Health for the treatment of vivax infection. Side effects include dizziness, skeletal muscle weakness, mild gastrointestinal disturbances (nausea, vomiting, abdominal discomfort, and diarrhea), and pruritus [26].

**Pharmacokinetics**

- **Artemether - Lumefantrine (Coartem):** artemether has a short t ½~ 1.5 hours, while lumefantrine has a long elimination half-life, 4 to 6 days
- **Chloroquine:** chloroquine has a long elimination half-life of 1-2 months.

**Drug Dosing and Regimens: All patients will be weighed to determine the accurate weight-based dose for both drugs.**

- **Artemether-lumefantrine** (Coartem; Novartis) administered twice daily for three days as tablets containing 20 mg of artemether plus 120 mg of lumefantrine in a fixed dose combination at a dosage [24].

| Weight (kg) | Age | Day 1 | | Day 2 | | Day 3 | |
| --- | --- | --- | --- | --- | --- | --- | --- |
|  |  | Morning | Evening | Morning | Evening | Morning | Evening |
| 5–14 | 3mo–2yrs | 1 | 1 | 1 | 1 | 1 | 1 |
| 15–24 | 3–7 yrs | 2 | 2 | 2 | 2 | 2 | 2 |
| 25–34 | 8–10 yrs | 3 | 3 | 3 | 3 | 3 | 3 |
| > 35 | 10+ yrs | 4 | 4 | 4 | 4 | 4 | 4 |

- **Chloroquine:** Total of 25mg base per kg over 3 days (10 mg base/kg on Days 1 and 2, and 5 mg base/kg on Day 3) [24]. 500 mg tablets and 10mg/ml syrup formulations. We will use the syrup formulation for children < 4 years of age.

| Weight (kg) | Age | Day 1 | Day 2 | Day 3 |
| --- | --- | --- | --- | --- |
| 5–6 | <4 mo | ¼ tablet  (5 ml syrup) | ⅛ tablet  (5 ml syrup) | ⅛ tablet  (2.5 ml syrup) |
| 7–10 | 4–11 mo | ¼ tablet  (7.5 ml syrup) | ¼ tablet  (7.5 ml syrup) | ¼tablet  (5 ml syrup) |
| 11–14 | 1–2 yrs | ½ tablet  (12.5 ml syrup) | ½ tablet  (12.5 ml syrup) | ¼ tablet  (7.5 ml syrup) |
| 15–18 | 3–4 yrs | ½ tablet  (15 ml syrup) | ½ tablet  (15 ml syrup) | ½ tablet  (15 ml syrup) |
| 19–24 | 5–7 yrs | ¾ tablet  (20 ml syrup) | ¾ tablet  (20 ml syrup) | ½ tablet  (15 ml syrup) |
| 25–35 | 8–10 yrs | 1¼ tablet | 1¼ tablet | ½ tablet |
| 36–50 | 11–13 yrs | 1½ tablet | 1½ tablet | 1 tablet |
| > 50 | 14+ yrs | 2 tablet | 2 tablet | 1 tablet |

**Intervention or treatment**

**Antimalarial Therapy-** All Pf patients enrolled into the study will be given AL while all Pv patients will be assigned to a drug group (AL or CQ) using a random number table. All drugs administered in the facility will be under supervision by the staff. Patients will be observed for 60 minutes after treatment for adverse reactions or vomiting. Those patients vomiting their medication within the first 30 minutes will receive a repeat full dose, those vomiting from 30-60 minutes will receive half dose. This is standard practice for anti-malarial treatment.

**Rescue Treatment**

Patients failing in any arm will be treated with quinine 10mg/kg/dose every 8 hours for 7 days as per National Guidelines [24]. This treatment will be administered orally unless the patient has persistent vomiting, in which case he/she will receive IM/IV therapy until able to tolerate oral therapy.

**Concomitant Treatment** Using standard clinic procedures, clinic personnel will administer supportive treatment to patients as necessary:

- **Antipyretics** will be given for temperatures > 39^o^C as per national policy. All patients enrolled in the study will be given two additional doses of paracetamol for use at home. Patients/parents/guardians should be instructed in the use and application of tepid sponging and fanning as per national policy.
- **Ferrous Sulfate/ Folate** will be given to all children with hemoglobin <10 mg/dl as per Integrated Management of Childhood Illness (IMCI) Guidelines
- **Anti-helminthics** will be given to children with hemoglobin <10 mg/dl over one year of age as per IMCI Guidelines

### Population

**Description and source of population and catchment area** The population of interest consists of patients aged above 6 months living in malarious areas diagnosed with uncomplicated *P. falciparum* or *P. vivax* malaria consulting at the health facility, who or whose parents or guardians give permission for study inclusion. For logistical reasons, only patients living near the enrolling health care facilities will be included (within 20 km), as we plan to visit the houses of those patients who do not return for scheduled follow-ups.

**Initial screening *inclusion* criteria:**

- Slide-confirmed infection with *P. falciparum*, with parasitemia of 1,000-100,000 asexual forms/ μl or slide confirmed infection with *P. vivax* with > 250 asexual forms/ μl
- Lives within 20 km of the enrolling health facility

**Inclusion criteria for enrollment:**

1. Meet screening inclusion criteria
2. Weight ≥ 5.0 kg
3. Axillary temperature ≥ 37.5º C or history of fever during the previous 24 or 48 hours for *P. falciparum* and *P. vivax* infection, respectively
4. Patient or caregiver agrees to all blood draws and return visits.

**Exclusion criteria for enrollment:**

1. General danger signs or symptoms of severe malaria (see Annex III)

2. Signs or symptoms of severe malnutrition, defined as weight-for-age ≤ 3 standard deviations below the mean (NCHS/WHO normalized reference values; see Annex IV)

3. Slide confirmed infection with any other *Plasmodium spp.* besides falciparum/vivax or mixed plasmodium infection

4. Severe anemia, defined as Hg < 5 g/dl

5. Known hypersensitivity to any of the drugs being evaluated

6. Presence of febrile conditions caused by diseases other than malaria

7. Serious or chronic medical condition (cardiac, renal, hepatic diseases, sickle cell disease, HIV/AIDS)

8. Pregnant or breastfeeding women.

9. Children weighing less than 5 kilograms.

**Justification of exclusion criteria**

Patients with history of danger signs, physical findings of severe illness or life-threatening anemia will be excluded and diverted for immediate management of their conditions. Those with non-malarial febrile illness in addition to malaria or mixed Plasmodium infections will be excluded because this will interfere with our ability to correctly classify the response to therapy. Patients with known sensitivity to drugs must be excluded and treated with an acceptable alternative per national guidelines. Those who plan to leave the study area during the follow-up period or living more than 20 km from the health center will also be excluded so as to minimize loss to follow-up and/or incomplete evaluation and treatment. Because we intend to follow participants at home in the event that they do not present for scheduled follow-up appointments, we need to restrict enrollees to those who live within 20 km of the health facility. Children less than 5 kilograms and pregnant or breastfeeding women will be excluded because there is limited data on drug dosing and safety in those groups.

**Sampling, sample size and statistical power**

i. Sample size determination:

With treatment failure of AL for either Pv or Pf and CQ for Pv in Ethiopia being reported at ≤5%, 10% has been chosen as the estimated therapeutic failure rate of the drugs. Therefore, we assumed an estimated ACPR of 90% for each drug investigated when determining sample size. Allowing for a 20% loss to follow-up at 42 days, a sample size of 120 subjects for each treatment arm will result in a 95% exact binomial confidence interval from 82.4%-95.1%.

# SCREENING AND ENROLLMENT PROCEDURES

**Screening Procedures for Enrollment**

Screening for eligible patients at the three study sites will be done in the following fashion:

1. All patients presenting to Adama Malaria Training Center and Debrezeit Malaria Center will be registered. All patients routinely have microscopy performed prior to clinical evaluation by a laboratory technician. Patients aged over 6 months with microscopy confirmed Pf or Pv who live within 20 km of the health facility will be screened.

2. All patients with temperature ≥37.5^o^C or a history of fever in the last 48 hours will have screening blood smear performed as per the national diagnosis and treatment guidelines at Awash Melkasa Health Center. Patients with microscopy confirmed Pf or Pv, age greater than 6 months, and living within 20 km of the health center will be screened.

3. For all facilities, if the patient meets the screening criteria and the patient or the parent or guardian asked for permission consents to enrollment, they will be assigned a consecutive, unique number that will become the patient's study number and will be used to identify all forms and blood samples from that patient. Enrollment will be conducted by a staff member not involved in the clinical assessment of the participant to ensure that consent is freely given.

4. A screening form containing demographic, clinical, and laboratory information will be started on each smear positive patient, thus allowing for appropriate tracking of each patient through the screening procedure.

5. The patient’s body weight will be measured, using a hanging scale for weighing young children, or a floor scale for large children/ adults and recorded on the demographic form.

**Enrollment Procedures**

If a patient meets the initial screening criteria and permission to participate in the study is obtained, the patient will go through the enrolling process.

1. Patient will be evaluated by clinical staff for signs of febrile illness other than malaria, (including but not limited to pneumonia, otitis media, tonsillitis, measles, chicken pox, abscesses) and signs of severe disease/ danger signs, as presence of any of these will exclude the patient from participating in the study. Patients found to have any of these conditions will be treated appropriately per National Guidelines, and referred to the referral hospital as necessary. A standardized form listing demographic and clinical information will be completed on each patient.

2. Urine pregnancy test will be conducted on all women aged 13-49. Anyone testing positive will be excluded.

3. Those enrolled will have a second finger prick performed to assess for the following:

● Thin smear will be used to verify parasite species and to conduct a formal parasite count. Two microscopists will independently read each smear (one at the study site and the other at Adama Reference Laboratory); if the 2 readings are >20% discordant, a third expert microscopist at the Oromia Regional Laboratory will examine the slide and make the final decision.

- Hemoglobin measurement (Hemocue™ Hb201+; HemoCue, Angelholm, Sweden) will be carried out and those with hemoglobin <5g/dL will be excluded.
- Filter paper will be obtained for molecular testing and drug levels.

4. Consecutive patients testing positive for Pf will be enrolled and treated with AL if they meet all inclusion criteria. Patients testing positive for Pv will be randomized to either CQ or AL treatment arms. A permuted block randomization technique will be used to ensure equal numbers in the two treatment arms. The study will not be blinded, although microscopists reading blood films will not be aware of which arm a participant is from.

5. After assignment, on day 0, each patient will receive the morning dose of treatment medication under direct observation and will be monitored for vomiting. The patient or parent will be given the evening dose (for AL only) to administer at home. Each patient will return on days 1 and 2 for subsequent doses. Clinical reassessments will be made on days 1- 3, 7, 14, 21, 28, 35, and 42 (see Annex V for a table of scheduled procedures). Participants will be given 35 Ethiopian Birr for each visit to cover their travel expenses (this is enough to cover round-trip transportation by bus to the clinic for one adult; parent traveling with the enrolled child requiring their own seat (age >6 years) will receive 50 Birr). Patients will be advised to return on *any* day during the follow-up period if symptoms return and not to wait for scheduled visit days. Patients who do not present to the facility for follow-up visits will be followed at home. This is standard for in vivo studies based on the WHO protocol, but is not the standard for routine care. Typically, for patients not enrolled in a study, the parent would be given all the doses of the medicine to administer at home, and no follow-up would be scheduled. The parent would be told to return if the child did not improve.

6. Blood films (thick and thin) for parasite count will be obtained and examined at each visit except day 1 and on any other day if the patient spontaneously returns with fever or worsening symptoms. Hemoglobin status will be measured on days 0, 7, 14, 21, 28, 35, and 42. A filter paper blood spot will be collected on days 0 and in case of treatment failure. All molecular testing will be conducted in Addis Ababa, Ethiopia, by Ethiopia Health and Nutrition Research Institute (EHNRI). Blood drug level testing capacity does not currently exist in Ethiopia and will be conducted at CDC Atlanta. All samples will have the study ID number and date of collection, but no other personal identifiers. The logbook that links the study ID number to the patient will be kept at the study site locked in a cabinet when not in use during the study enrollment period. After the completion of the study, this logbook will be stored in a locked cabinet in Addis Ababa at the International Center for AIDS Care and Treatment Program’s office.

# FOLLOW-UP PROCEDURES

**Study Instruments**

Standard operating procedures and structured forms for clinical history, physical assessment, and data collection in English will be used by each study site. All study staff are familiar with English and health systems data collection in the country are routinely done in English.

# END-POINTS

**Outcomes and minimal differences**

The primary outcome of this study will be to estimate the efficaciousness of artemether-lumefantrine against falciparum malaria and vivax malaria and chloroquine against vivax malaria.

The classification of treatment outcomes will be based on an assessment of the parasitological and clinical outcome of antimalarial treatment according to the latest guidelines from WHO. Accordingly, all Pf patients will be classified as having an Early Treatment Failure (ETF), a Late Clinical Failure (LCF), a Late Parasitological Failure (LPF), or an Adequate Clinical and Parasitological Response (ACPR).

**Classification of Treatment Outcomes, WHO 2003**

**Early Treatment Failure (ETF)**

- Development of danger signs or severe malaria on day 1, day 2 or day 3 in the presence of parasitemia
- Parasitemia on day 2 higher than day 0 count irrespective of axillary temperature
- Parasitemia on day 3 with axillary temperature ≥37.5 ºC
- Parasitemia on day 3 ≥25% of count on day 0

**Late Treatment Failure (LTF)**

***Late Clinical Failure* (LCF)**

- Development of danger signs or severe malaria after day 3 in the presence of parasitemia, without previously meeting any of the criteria of Early Treatment Failure
- Presence of parasitemia and axillary temperature ≥37.5 ºC or history of fever on any day from day 4 to day 28 or 42, without previously meeting any of the criteria of Early Treatment Failure

***Late Parasitological Failure* (LPF)**

- Presence of parasitemia on any day from day 7 to day 28 or 42 and axillary temperature <37.5 ºC, without previously meeting any of the criteria of Early Treatment Failure or Late Clinical Failure

**Adequate Clinical and Parasitological Response (ACPR)**

- Absence of parasitemia on day 28 or 42 irrespective of axillary temperature without previously meeting any of the criteria of Early Treatment Failure or Late Clinical Failure or Late Parasitological Failure

In order to differentiate a recrudescence (same parasite strain) from a newly acquired infection (different parasite strain), a genotypic analysis based on merozoite surface protein-1 (*msp1)*, merozoite surface protein 2 (*msp2)* and glutamate-rich protein (*glurp)* will be performed by PCR for Pf. Additionally, we will characterize microsatellite markers for Pf to differentiate recrudescence from reinfection and potentially assess for drug resistance markers.

**Definition of Treatment Outcomes for *P. vivax* infections, WHO 2002** [4]

Treatment failure defined as:

1. Clinical deterioration due to *P. vivax* illness requiring hospitalization in presence of parasitemia
2. Presence of parasitemia and axillary temperature ≥37.5^o^C any time between Day 3 and Day 28 or 42
3. Presence of parasitemia on any day between day 7 and day 28 or 42, irrespective of clinical conditions.

*P. vivax* infections that recur after drug treatment can have 1 of 3 etiologies: 1) recrudescence, 2) reinfection, or 3) relapse arising from the dormant liver stage (hypnozoites). In order to differentiate the treatment failures as recrudescence or relapse from a newly acquired infection, a genotypic analysis based on P. vivax circumsporozoite protein (*pvcs*), the merozoite surface protein 1 (*pvmsp1*) and the merozoite surface protein 3-alpha (*pvmsp3-alpha*) will be performed by PCR for Pv. Additionally, we also will characterize microsatellite markers to assess recrudescence from reinfection and for drug resistance markers. Those who fail in the vivax arms will have drug levels tested from the already collected filter paper.

**Valid end-points** include treatment failure, completion of the follow-up period without treatment failure, loss to follow-up, withdrawal from study (voluntary or involuntary, and protocol violation). Loss to follow-up occurs when, despite all reasonable effort, an enrolled patient who does not attend the scheduled visits, cannot be found. Patients who withdraw from the study during the treatment period (days 0-2) will be treated with AL or CQ depending on the species according to the national policy. They will be referred back to the clinic to continue with care.

**Criteria for withdrawal**

Patients meeting any of the following criteria will be withdrawn:

- Withdrawal of consent
- Failure to complete the treatment
- Vomiting both initial and replacement doses at any single time in the treatment (i.e. If the patient vomits both attempts to administer the morning dose that would require withdrawal; however, vomiting the initial morning dose but not the morning replacement dose would not require withdrawal), persistent vomiting
- Severe side-effects necessitating hospitalization
- Progression to severe malaria
- Occurrence during the follow-up of concomitant disease that would interfere with a clear classification of the treatment outcome
- Antimalarial (or antibiotics with antimalarial activity) treatment administered by a third party or self-medication with antimalarial (or antibiotics with antimalarial activity) assessed by asking participants or their caretaker at follow-up visits
- Detection of another malaria species infection during the follow-up
- Erroneous inclusion of a patient outside of the inclusion/exclusion criteria
- Misclassification of a patient due to a laboratory error (parasitemia) leading to the administration of the rescue treatment

**Training for personnel**: Clinicians and laboratory personnel are trained and licensed in Ethiopia. In addition, they will receive approximately 1 week additional training to review the protocol of this study. In addition to technical aspects of specific laboratory techniques and clinical assessments, training will stress the importance of confidentiality, appropriate techniques for obtaining informed consent, Staff will participate in practical exercises. Continued appropriate behavior with regard to informed consent will be reinforced by intermittent observation by the supervisor. The staff will include 1-2 laboratory technicians for microscopy, and 1-2 staff persons for enrollment and clinical assessments.

# DATA HANDLING AND ANALYSIS

**Data analysis plan, including statistical methodology and planned tables and figures**

SAS 9.1 (Cary, NC) as well as the WHO drug efficacy tools provided at <http://www.who.int/malaria/toolsformonitoring.html> will be used for data management and analysis. Data will be analyzed using two methods: **per protocol analysis**, where patients who were withdrawn from the study or who were lost to follow-up are excluded, and **survival analysis**, where all enrolled patients are included in the analysis until the last day before drop-out. In addition to voluntary withdrawals or treatment non-compliance, patients will be considered for the analysis as withdrawn if the PCR results are unclassifiable.

The final analysis will include:

- A description of the patients screened, and the distribution of the reasons for exclusion.
- A description of the patients included in the study.
- The proportion of patients lost to follow-up or withdrawn and the distribution of reasons for withdrawal.
- The PCR-adjusted and unadjusted proportion of ETF, LCF, LPF, and ACPR in the Pf arm at day 28 and day 42 with 95% confidence intervals using both methods of analysis.
- Both drug-level and PCR-adjusted and unadjusted proportion of treatment failures in each Pv arm at day 28 and day 42 with 95% confidence intervals using both methods of analysis
- Frequency of side effects, severe malaria, anemia, and hospitalizations by treatment arm

**Data collection**: Data will be collected and recorded at the health facility by one of the trained staff members. All data entry forms will be checked for completeness. Laboratorians may complete brief reports on laboratory findings to share with clinicians who will transfer these results to the case report form of the appropriate patient, the laboratory results will be entered into an Excel database.

**Information management and analysis software:** Microsoft Excel (Redmond, Washington) will be used for double entry of laboratory data and clinical data. SAS 9.1 (Cary, NC) will be used for data analysis.

**Data entry, editing, handling, storage and disposition**: Some open-ended data including the names of specific drugs or diagnoses will be coded prior to data entry. A list of standard codes for these fields will be developed by hand once collection of data is complete. All forms will then be coded and checked by 2 different team members.

Data will be double entered using Microsoft Excel (Redmond, Washington). Systematic routines will be developed to check for data entry discrepancies and range and consistency. All discrepancies will be resolved by reference to the original checked data collection forms. The final data set will be transformed to SAS for analysis and will no longer contain any personal identifying information.

Original data collection forms will be handled only by staff members and kept under locked storage until completely coded, checked and transported for data entry. Once data entry and cleaning are complete the original forms will be stored under lock and key at ICAP until final analyses and reports have been prepared. They will then be destroyed.

**Storage of specimens**: Specimens obtained in this study will be saved only for the duration of molecular testing and drug testing of treatment failures and then destroyed. No samples will be stored. Specimens will not be tested for HIV.

**Quality control/ assurance**: Site supervisors will check data collection forms at the end of follow-up for completeness and accuracy of recording.

**Bias in data collection, measurement and analysis**: Some bias may be introduced through the fact that both treating clinicians and parents will be aware of which treatment a subject received; as much as possible we will rely on objective measures of improvement (fever resolution, malaria parasitemia) which will be unaffected by knowing the treatment allocation. In addition, the microscopists reading the slides will be blinded to the treatment allocation. Other biases in data collection will be minimized by use of standardized data collection instruments.

**Intermediate reviews**: Although this study aims to study the efficacy of accepted and widely used first-line treatments in Ethiopia, a data safety monitoring board (DSMB) consisting of members not involved in the study will be set up to protect the rights and welfare of the subjects. The objectives, accountability, responsibilities, membership, and procedures of the DSMB are detailed in Annex VIII.

**Limitations**: Results from this study may not be applicable to other ACTs or other areas of Africa with different resistance patterns. In addition, the nature of *in vivo* drug efficacy studies always involves some compromise between statistical power, representativeness, and practical logistic constraints. Therefore the study will not be powered to estimate differences in efficacy across treatment arms or between sites. The three health facilities were not chosen randomly and thus may not be representative of our target population.

# HANDLING OF UNEXPECTED OR ADVERSE EVENTS

**Response to new or unexpected findings**

All treatment failures will be treated with quinine as per the National Guidelines. If sufficient number of malaria cases are presenting to the clinic but enrolment is insufficient, the supervisor will need to determine at what point the problem with enrollment is occurring, and take steps needed to rectify the problem. If the problem is not remedied, the supervisor will report the poor enrollment to the investigators at CDC, Atlanta, and steps will be taken to either rectify the problem or to find a new site.

**Identifying, managing and reporting adverse events**

The combination of artemether + lumefantrine has been shown to be safe and highly efficacious [25]. Studies of all of these combinations have reported only mild or moderate adverse events, including dizziness, headache, anorexia, nausea and abdominal pain, noting that these events may also be attributable to malaria.

Severe allergic reactions have not been reported in association with artemisinin use. Anaphylactic type reactions to artemisinins have been reported very infrequently; these are estimated to occur in 1 in 2833 patients who receive an artemisinin (95% confidence interval 1 in 1362 to 6944; 6/ 17,000 patients) [27]. There has been one additional report of angioedema suspected to be due to the artemether component of artemether-lumefantrine [28]. With the initial development of AL, its safety was compared to CQ in the treatment of falciparum malaria. An efficacy study in Tanzanian children reported no serious adverse events in either group [29]. Two other studies comparing AL with CQ for the treatment of falciparum malaria noted more frequent reports of pruritus and vomiting in the CQ group [30, 31]. We will be observing all the children for an hour following the ingestion of every dose of antimalarial. Most severe allergic reactions will manifest within an hour of taking the dose. We will council patients and parents to return immediately if they or their child develops a raised rash (urticaria) or difficulty breathing suggestive of a severe allergic reaction.

In the event of mild adverse reactions, it will be noted in the subjects case report form that these occurred; no further action will be taken by staff members, except in the case of vomiting, in which case the treatment medication may need to be re-administered.

In the event of significant vomiting, initially ORS (oral rehydration solution) will be administered, if this is not tolerated the patient will be referred to the district hospital for further management. In the case of any severe adverse reaction (difficulty breathing, convulsions, and change in mental status) subjects will be referred to the district hospital for management. Transportation to the hospital will be provided.

Any adverse event which is severe, unexpected, and possibly related to the study, as well as any unanticipated problems involving risks to subjects or others and any occurrences of serious or continuing non-compliance will be reported to the CDC IRB according to CDC policy on incident reporting.

**EMERGENCY CARE**

Any patient with treatment failure or recrudescence will receive quinine 10mg/kg/dose every 8 hours for 7 days. This will be given orally unless the patient has persistent vomiting, in which case it will be given IM/IV until the vomiting resolves and then will be switched to oral therapy.

Patients with signs of severe malaria (altered mental status, coma, convulsions, hemoglobin <5g/dl, Respiratory distress, circulatory collapse, or abnormal bleeding), persistent vomiting or severe side effects will be hospitalized and receive parenteral therapy with quinine and relevant supportive treatments, according to National Guidelines.

Any participant who develops urticaria or difficulty breathing following administration of the treatment drug will be treated with diphenhydramine or chlorpheniramine and will be withdrawn from the study. In addition, patient and or their parents, caretakers and family members will be counseled that the patient should not receive additional doses of the antimalarial they were given. These patients will be treated with oral quinine for seven days as per the rescue protocol.

Adult patients and parents with children in the study will be encouraged to return to the clinic for any fever or other signs of illness on any day during the 42 day follow-up period, allowing for early detection and treatment of illness before it becomes severe.

Any hospitalization which occurs during the 42 day follow-up period will be paid for by the study.

# ETHICAL ISSUES

**Consent Process**

Staff members will fully explain the assessments to the participants and or their caretakers and ask for their consent/assent to participate. The written consent documents in English are attached as Annex VII. These will be translated in Afaan Oromoo and Amharic and then back translated to verify accuracy. These will be read to participants and or their caretakers in their respective local language. If a participant or parent/ guardian are illiterate, the consent form will be read to them in their respective local language and a thumbprint will be accepted as a legally effective signature. Consenting participants and or their caretakers will be advised that they are free to decline any question or procedure and that they may terminate their participation at any time without loss of any benefits.

**Purpose.**  The clinical efficacy of the first-line antimalarials CQ and AL will be assessed using a WHO standardized procedure. These results will be used to assist Ethiopia in re-assessing their national malaria drug policies and investigating therapeutic options for the future.

**Procedures**. During the course of the study, patients will receive an antimalarial and will undergo 9-10 finger pricks to allow monitoring of parasitemia and anemia. Clinical assessments will be made on days 0, 1- 3, 7, 14, 21, 28, 35, and 42.

**Duration of participant involvement.** Patients will be involved in the study from day of enrollment (Day 0) until their 42 day follow-up.

**Experimental products or procedures.** No experimental therapies, products, or procedures will be used in this study. The use of AL for the treatment for Pv is common practice and WHO recommended practice in countries that have adopted AL for first-line treatment of Pf.

**RISKS**

There is minimal physical and psychological risk associated with this study. The patient may experience a brief moment of physical discomfort and/or fear during the blood drawing procedure. There will be blood draws collected by finger pricks.

There is minimal risk associated with taking the treatment drugs. All of the drugs are recommended therapies in Ethiopia, have been widely used, and have been found to be safe. Studies of all of these combinations have reported only mild or moderate adverse events, including dizziness, headache, anorexia, nausea and abdominal pain, noting that these events may also be attributable to malaria.

There are no social risks associated with this protocol. Malaria is a widely recognized disease in Ethiopia, and seeking treatment is socially acceptable. There are no economic risks associated with this study. Patients will be assessed and treated free of charge, and families will be given a stipend of 35 Ethiopian Birr (50 Birr for those accompanied by a parent) per visit to cover their travel expenses. Any hospitalization that results from this study or during the follow-up period will be paid for.

**METHODS TO MINIMIZE RISKS**

In order to minimize risks to patients, subjects will be followed closely for signs of recurrent or recrudescent parasitemia, with clinical exams on days 1- 3, 7, 14, 21, 28, 35, and 42 and malaria smears on all those days except day 1. In addition, patients will be encouraged to return on any other day with signs of illness or fever. All patients will be observed closely for an hour following the first dose of medication; if this is well tolerated, participants will be given the option of taking the evening dose home with them or returning to the clinic in the evening for administration of the drug. Any patient who develops urticaria or difficulty breathing following administration of the treatment drug will be treated with diphenhydramine or chlorpheniramine and will be withdrawn from the study. In addition, participants will be counseled that they should not take additional doses of other antimalarials.

**ANTICIPATED BENEFITS**

This study is of direct benefit to participants. All patients will be treated for their initial parasitemia and any additional parasitemia that presents with clinical symptoms. Any patient failing treatment with the treatment drug regimen will be treated with quinine.

At each visit, the patient, parent or guardian will be informed as to the status of their or their child’s health, and the procedures and/or treatments that will occur during that visit and have the right to refuse a procedure or treatment or request transfer to the regular clinic and withdrawal from the study. All patient costs associated with the study will be paid for. Patients with malaria at government clinics receive for free whatever treatment is available in stock at the clinic. Although AL and CQ is the national policy, it is not always available in government clinics. Additionally, patients typically receive their medication and are discharged without further follow up. Participants would have both clinical and laboratory analysis on the prescribed days to ensure clinical resolution and clearance of parasitemia. Transportation and hospitalization during that 42-day period for other illnesses or for outpatient treatment failure will be free of charge. The participants would have to be in the study to receive these additional benefits.

The findings from the study will assist decision making to improve prevention and treatment of malaria in Ethiopia. With universal access to AL in Ethiopia since 2004 and the long-term use of chloroquine for Pv, monitoring for resistance to both these first-line drugs will be paramount. As a country with two first-line drugs, they are currently discussing the use of AL as the first-line treatment for all malaria regardless of the species. Prior to making this costly shift with little empiric evidence, the efficaciousness of AL for Pv needs to be further assessed.

**RISK/BENEFIT RATIO**

This study has minimal risks and significant benefit to the enrolled subjects.

**VULNERABLE POPULATIONS**

**Children**- This minimal risk study includes children. Because this group has minimal natural immunity, this is the group most at risk for severe malaria, so any treatment must be efficacious in this age group. Adequate provisions are made for soliciting the permission of a parent or guardian.

**Non-English speaking participants**- It is imperative to carry out this study in the areas in which malaria is endemic; therefore, it is unavoidable that some participants will be non-English speaking. All consent forms have been translated into Afaan Oromoo and Amharic and back translated into English for accuracy; all explanations of the study procedures and clinical findings will be carried out in the patient’s local language by local staff member.

**INFORMED CONSENT/ PARENTAL PERMISSION PROCESS/ DOCUMENTATION OF INFORMED CONSENT**

Permission will be obtained from parent/ guardian bringing the child or the adult him/herself. Consent, and assent for children 7-17 years of age, will be appropriately documented prior to each patient’s enrollment. As this study is minimal risk, permission from only one parent will be required. A staff member will read the form to the patient and or parent and give them a chance to ask questions. Patients and or parents who are able to sign their name will be asked to sign the consent form. For non-literate persons, a thumbprint will be obtained in lieu of a signature. Children age 7-11 years of age will have a simplified assent form read to them. Details about the study and its benefits and potential risk will be explained to the patients in the language in which they are most fluent. All information regarding the patients will remain confidential to the extent legally possible. Unique numerical identifiers will be used for data entry.

**JUSTIFICATION FOR WAIVER/ ALTERATION OF INFORMED CONSENT**

No justification for waiver or alteration of informed consent is being sought.

There is a large illiterate population in Ethiopia; thus there is a high likelihood that parents may be unable to sign their names. In this case, permission will be documented with a thumbprint as a legally effective signature in Ethiopia, following thorough explanation of the study and its benefits and potential risk to the patients and or parents/guardians in the language in which they are most fluent. A witness who is not involved with enrolling the patients and literate will witness and sign the informed consent.

**ASSENT IMPLEMENTATION PROCESS/ DOCUMENTATION OF ASSENT**

Assent for children age 7-17 years of age will be sought.

**PROTECTION OF PRIVACY AND CONFIDENTIALITY**

**Privacy of individual** Examinations will take place in a private room. Individual data will be reported to the participants and to relevant staff members, and will be recorded in the health card.

**Confidentiality of data** All screening forms and case record forms will be kept in a secured location with access limited to authorized staff members. Unique numerical identifiers will be used for the computer-based data entry and blood samples. Publications will contain only aggregate data. No identifying information will be included.

**EXTRA COSTS TO PARTICIPANTS**

Participants will not incur extra costs through their participation in this study. All examinations, laboratory testing, and medications will be provided free. Participants will be reimbursed for their travel costs. Their participation in the study will require additional visits, so there will be an extra time cost associated with participation.

**REIMBURSEMENTS OR INCENTIVES**

Patients will be reimbursed 35 Ethiopian Birr (50 Birr those children age>6 years accompanied by a parent) per visit for their travel expenses to minimize loss to follow-up and to prevent patients from incurring extra expenses in travel to and from the clinic for the purpose of participating in the study. The travel reimbursement will be given to each participant even if they are in the same family.

# DISSEMINATION, NOTIFICATION AND REPORTING OF RESULTS

**Notifying participants of their individual results**

Participants and their parents will be informed of the results of the malaria smear, hemoglobin level, and clinical assessments at the time of enrollment and at each follow-up visit. Filter paper analysis will be completed only after the study is completed at a reference laboratory. Participants will not be informed of their individual results from these tests.

**Notifying participants of findings**: Participants will not be directly notified of the study findings.

**Anticipated products or inventions resulting from the study and their use**

All results will be disseminated to the national malaria control programs and other appropriate bodies within the Ethiopian Government. We anticipate that the results of this study will be used to inform future changes to the National Malaria Diagnosis and Treatment Guidelines of Ethiopia. In addition, it is anticipated that the evaluators will develop abstracts for presentation at scientific meetings. Finally, one or more detailed manuscripts will be prepared for publication in a peer-reviewed scientific journal.

**Disseminating results to public**

The Ethiopian FMOH regularly disseminates key findings to the general public. This occurs through press releases and by including media reporters in data dissemination workshops with local leaders at the conclusion of each major activity, or sooner as important findings become available. Findings from this proposed study that may be of value to the general public can be disseminated through these existing mechanisms.

**Scientific Peer Review**

The results from this study will be published in at least one summary paper which will be submitted to a peer-review journal. In addition, additional abstracts may be developed from the results of this study.

**Conflicts of Interest**

We have no conflicts of interest.

# REFERENCES

1. WHO, *World Malaria situation in 1993, part I.* Weekly Epidemiological Record, 1996. **71**(3): p. 17-22.

2. WHO, *World Malaria Report 2008*. 2008, World Health Organization: Geneva.

3. Bloland, P.B., et al., *Beyond chloroquine: implications of drug resistance for evaluating malaria therapy efficacy and treatment policy in Africa.* J Infect Dis, 1993. **167**(4): p. 932-7.

4. WHO, *Monitoring Antimalarial Drug Resistance: Report of a WHO consultation*. 2002, World Health Organization: Geneva.

5. WHO, *Assessment and Monitoring of Antimalarial Drug Efficacy for the Treatment of Uncomplicated Falciparum Malaria*. 2003, World Health Organization: Geneva.

6. Nigatu, W., M. Abebe, and A. Dejene, *Plasmodium vivax and P. falciparum epidemiology in Gambella, south-west Ethiopia.* Trop Med Parasitol, 1992. **43**(3): p. 181-5.

7. Ramos, J.M., F. Reyes, and A. Tesfamariam, *Change in Epidemiology of Malaria Infections in a Rural Area in Ethiopia.* J Travel Med, 2005. **12**: p. 155-156.

8. Fogh, S., S. Jepsen, and R. Mataya, *Chloroquine-resistant Plasmodium falciparum malaria from Kenya.* Trans R Soc Trop Med Hyg, 1979. **73**: p. 228-229.

9. Alene, G.D. and S. Bennett, *Chloroquine resistance of Plasmodium falciparum malaria in Ethiopia and Eritrea.* Trop Med Int Health, 1996. **1**(6): p. 810-5.

10. Kebede, F., N. Taffa, and T. Tedla, *An in-vivo study of falciparum malaria sensitivity to Chloroquine in unstable malaria endemic area of central Ethiopia.* Ethiop Med J, 1999. **37**(2): p. 97-109.

11. Tulu, A.N., et al., *Failure of chloroquine treatment for malaria in the highlands of Ethiopia.* Trans R Soc Trop Med Hyg, 1996. **90**(5): p. 556-7.

12. Abebe, W., *Therapeutic efficacy of sulfadoxin/pyrimethamine in the treatment of uncomplicated Plasmodium falciparum malaria in Enseno, Meskan Woreda, Gurage zone, SNNPR, Ethiopia.* Ethiop Med J, 2006. **44**(2): p. 133-8.

13. Degefa, T., *In vivo sulphadoxine-pyrimethamine sentitivity study Tigray Region, Southern Zone, Alamata Town, September--November 2001.* Ethiop Med J, 2004. **42**(1): p. 35-9.

14. Kassa, M., et al., *Development of resistance by Plasmodium falciparum to sulfadoxine/pyrimethamine in Amhara Region, Northwestern Ethiopia.* Ethiop Med J, 2005. **43**(3): p. 181-7.

15. Jima, D., et al., *Safety and efficacy of artemether-lumefantrine in the treatment of uncomplicated falciparum malaria in Ethiopia.* East Afr Med J, 2005. **82**(8): p. 387-90.

16. Falade, C., et al., *Efficacy and safety of artemether-lumefantrine (Coartem) tablets (six-dose regimen) in African infants and children with acute, uncomplicated falciparum malaria.* Trans R Soc Trop Med Hyg, 2005. **99**(6): p. 459-67.

17. Guthmann, J.P., et al., *High efficacy of two artemisinin-based combinations (artesunate + amodiaquine and artemether + lumefantrine) in Caala, Central Angola.* Am J Trop Med Hyg, 2006. **75**(1): p. 143-5.

18. Makanga, M., et al., *Efficacy and safety of the six-dose regimen of artemether-lumefantrine in pediatrics with uncomplicated Plasmodium falciparum malaria: a pooled analysis of individual patient data.* Am J Trop Med Hyg, 2006. **74**(6): p. 991-8.

19. Mutabingwa, T.K., et al., *Amodiaquine alone, amodiaquine+sulfadoxine-pyrimethamine, amodiaquine+artesunate, and artemether-lumefantrine for outpatient treatment of malaria in Tanzanian children: a four-arm randomised effectiveness trial.* Lancet, 2005. **365**(9469): p. 1474-80.

20. WHO, *Guidelines for the Treatment of Malaria*. 2006, World Health Organization: Geneva.

21. Krudsood, S., et al., *Clinical efficacy of chloroquine versus artemether-lumefantrine for Plasmodium vivax treatment in Thailand.* Korean J Parasitol, 2007. **45**(2): p. 111-4.

22. Pukrittayakamee, S., et al., *Therapeutic responses to different antimalarial drugs in vivax malaria.* Antimicrob Agents Chemother, 2000. **44**(6): p. 1680-5.

23. Teka, H., et al., *Chloroquine-resistant Plasmodium vivax malaria in Debre Zeit, Ethiopia.* Malar J, 2008. **7**: p. 220.

24. Federal Ministry of Health Ethiopia, *Malaria: Diagnosis and Treatment Guidelines for Health Workers in Ethiopia*. 2004.

25. Maiteki-Sebuguzi, C., et al., *Safety and tolerability of combination antimalarial therapies for uncomplicated falciparum malaria in Ugandan children.* Malar J, 2008. **7**: p. 106.

26. Baird, J.K., *Effectiveness of antimalarial drugs.* N Engl J Med, 2005. **352**(15): p. 1565-77.

27. Leonardi, E., et al., *Severe allergic reactions to oral artesunate: a report of two cases.* Trans R Soc Trop Med Hyg, 2001. **95**(2): p. 182-3.

28. Krippner, R. and J. Staples, *Suspected allergy to artemether-lumefantrine treatment of malaria.* J Travel Med, 2003. **10**(5): p. 303-5.

29. Hatz, C., et al., *Efficacy and safety of CGP 56697 (artemether and benflumetol) compared with chloroquine to treat acute falciparum malaria in Tanzanian children aged 1-5 years.* Trop Med Int Health, 1998. **3**(6): p. 498-504.

30. Bakshi, R., et al., *An integrated assessment of the clinical safety of artemether-lumefantrine: a new oral fixed-dose combination antimalarial drug.* Trans R Soc Trop Med Hyg, 2000. **94**(4): p. 419-24.

31. Kshirsagar, N.A., et al., *A randomized, double-blind, parallel-group, comparative safety, and efficacy trial of oral co-artemether versus oral chloroquine in the treatment of acute uncomplicated Plasmodium falciparum malaria in adults in India.* Am J Trop Med Hyg, 2000. **62**(3): p. 402-8.

32. *Severe falciparum malaria. World Health Organization, Communicable Diseases Cluster.* Trans R Soc Trop Med Hyg, 2000. **94 Suppl 1**: p. S1-90.

# ANNEX

### ANNEX I *P. falciparum* treatment outcomes

| **Classification of treatment outcomes for falciparum malaria**  **WHO [5]** |
| --- |
| **Early Treatment Failure (ETF)** |
| - Development of danger signs or severe malaria on day 1, day 2 or day 3 in the presence of parasitemia; - Parasitemia on day 2 higher than day 0 count irrespective of axillary temperature; - Parasitemia on day 3 with axillary temperature ≥37.5 ºC; - Parasitemia on day 3 ≥25% of count on day 0. |
| **Late Treatment Failure (LTF)** |
| ***Late Clinical Failure* (LCF)**   - Development of danger signs or severe malaria after day 3 in the presence of parasitemia, without previously meeting any of the criteria of Early Treatment Failure; - Presence of parasitemia and axillary temperature ≥37.5 ºC or history of fever during the preceding 24 hours from any day between day 4 to day 42, without previously meeting any of the criteria of Early Treatment Failure.   ***Late Parasitological Failure* (LPF)**   - Presence of parasitemia on any day from day 7 to day 42 and axillary temperature <37.5 ºC, without previously meeting any of the criteria of Early Treatment Failure or Late Clinical Failure. |
|
| **Adequate Clinical and Parasitological Response (ACPR)** |
| - Absence of parasitemia on day 42 irrespective of axillary temperature without previously meeting any of the criteria of Early Treatment Failure or Late Clinical Failure or Late Parasitological Failure. |

### ANNEX II *P. vivax* treatment outcomes

| **Definition of treatment outcomes for vivax malaria**  **WHO [4]** |
| --- |
| **Treatment Failure (TF)** |
| - Clinical deterioration due to *P. vivax* illness requiring hospitalization in presence of parasitemia - Presence of parasitemia and axillary temperature ≥37.5^o^C any time between Day 3 and Day 28 or Day 42 - Presence of parasitemia on any day between day 7 and day 28 or day 42, irrespective of clinical conditions |

### ANNEX III Severe malaria definitions

Definition of severe malaria [32]

Severe manifestations of P. falciparum malaria in adults and children

| **Prognostic value^a^** | | | **Frequency^a^** | |
| --- | --- | --- | --- | --- |
| Children | Adults |  | Children | Adults |
| *Clinical Manifestations* | | | | |
| + | (?)^b^ | Prostration | +++ | +++ |
| +++ | + | Impaired Consciousness | +++ | ++ |
| +++ | +++ | Respiratory Distress (acidotic breathing) | +++ | + |
| + | ++ | Multiple convulsions | +++ | + |
| +++ | +++ | Circulatory collapse | + | + |
| +++ | +++ | Pulmonary edema | +/– | + |
| +++ | ++ | Abnormal bleeding | +/– | + |
| ++ | + | Jaundice | + | +++ |
| + | + | Hemoglobinuria | +/– | + |
| *Laboratory findings* | | | | |
| + | + | Severe anemia | +++ | + |
| +++ | +++ | Hypoglycemia | +++ | ++ |
| +++ | +++ | Acidosis | +++ | ++ |
| +++ | +++ | Hyperlactatemia | +++ | ++ |
| +/– | ++ | Hyperparasitemia | ++ | + |
| ++ | ++ | Renal Impairment | + | +++ |

^a^On a scale from + to +++; +/– indicates infrequent occurrence

^b^Data not available

### ANNEX IV Weight for Height Chart


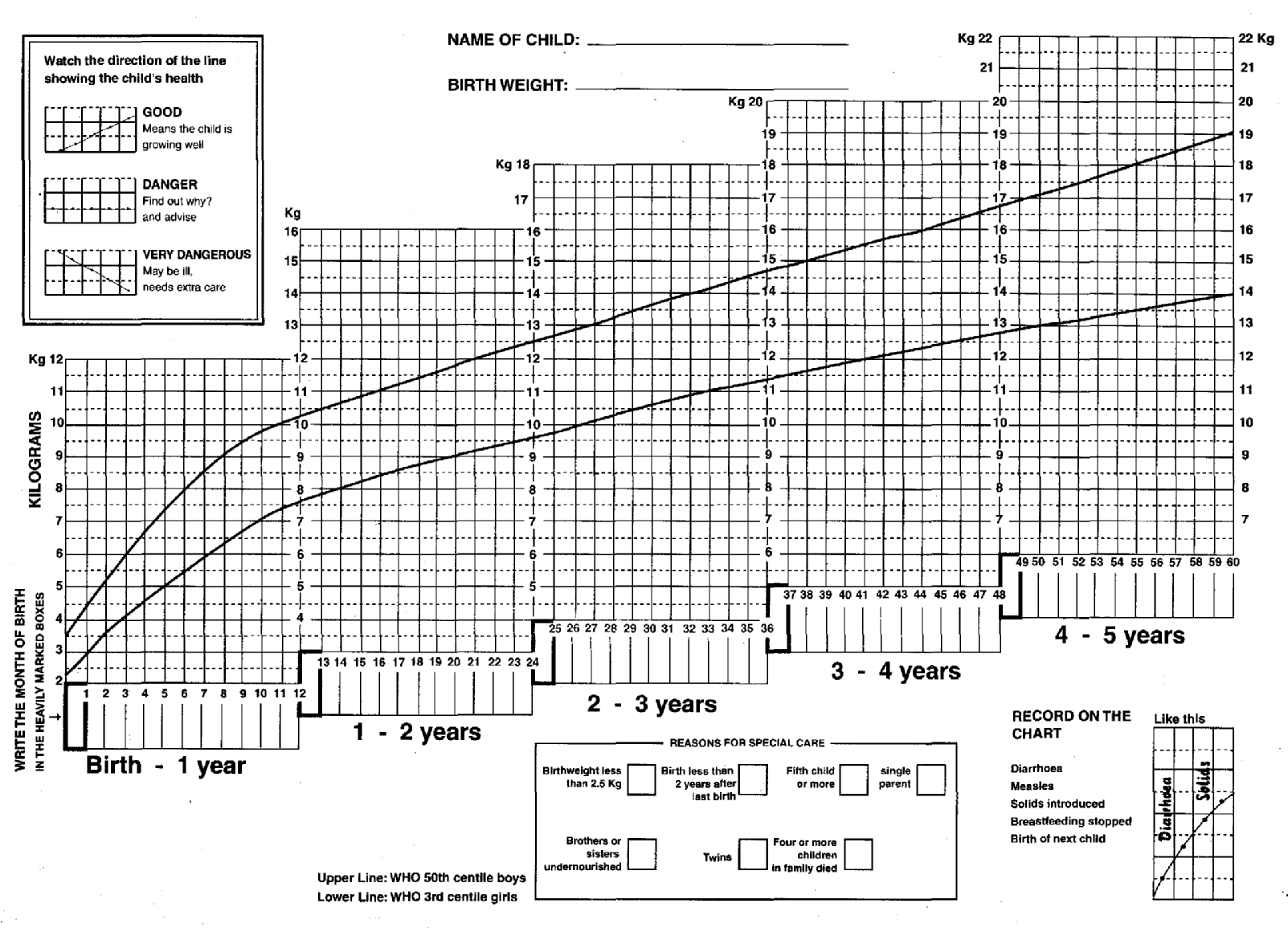


### ANNEX V Schedule of follow-up activities

|  | day 0 | day 1 | day 2 | day 3 | day 7 | day 14 | day 21 | day 28 | day 35 | day 42 |
| --- | --- | --- | --- | --- | --- | --- | --- | --- | --- | --- |
| PROCEDURES |  |  |  |  |  |  |  |  |  |  |
| Clinical assessment | X | X | X | X | X | X | X | X | X | X |
| Temperature | X | X | X | X | X | X | X | X | X | X |
| Blood slide for parasites count | X | (X) | X | X | X | X | X | X | X | X |
| Hemoglobin | X |  |  |  | X | X | X | X | X | X |
| Filter paper | X | (X) | (X) | (X) | (X) | (X) | (X) | (X) | (X) | (X) |
| TREATMENT |  |  |  |  |  |  |  |  |  |  |
| Drug to be given | X | X | X |  |  |  |  |  |  |  |
| Rescue treatment |  | (X) | (X) | (X) | (X) | (X) | (X) | (X) | (X) | (X) |

**NOTES:** Parentheses denote conditional or optional activities. On Day 1, the patient should be examined for parasitemia if she or he has any danger sign. Rescue treatment could be given on any day, provided that the patient meets the criteria for treatment failure. Extra days are any days other than regularly scheduled follow-up days when the patient returns to the facility because of recurrence of symptoms. On extra days, blood slides may or may not be routinely taken. Filter paper collection for PCR will be taken on day 0 and on day of treatment failure.

### ANNEX VI Study Schedule

**Day 0:**

Screening

Clinical assessment including measurement of weight and height — referral in case of severe malaria/danger signs

Measurement of axillary temperature

Parasitological assessment

Informed consent

Enrollment

Treatment, first dose

Hemoglobin, filter paper for PCR

**Day 1:**

Clinical assessment — referral in case of severe malaria/danger signs

Measurement of axillary temperature

Parasitological assessment

Treatment, second dose or alternative treatment in case of early treatment failure

**Day 2:**

Clinical assessment — referral in case of severe malaria/danger signs

Measurement of axillary temperature

Parasitological assessment

Treatment, third dose or alternative treatment in case of early treatment failure

**Day 3, Day 7, Day 14, Day 21, Day 28, Day 35, and Day 42:**

Clinical assessment — referral in case of severe malaria/danger signs

Measurement of axillary temperature

Parasitological assessment

Alternative treatment in case of treatment failure

Hemoglobin/hematocrit (Day 7, Day 14, Day 28, Day 42), filter paper sampling for PCR in case of treatment failure

**Any other day:**

Clinical assessment — referral in case of severe malaria/danger signs

Measurement of axillary temperature

Parasitological assessment

Hemoglobin/hematocrit if indicated clinically

Alternative treatment in case of treatment failure

Optional: blood sampling for PCR

### ANNEX VII Consent/Assent Form

### Informed consent form (for adult consent and parental permission for child six years and under), and assent form (for child 7-17 years), for enrolment for malaria in vivo efficacy study (Flesch-Kincaid Reading Level 8.9)

**Contact Person:**

**Dr. Zenebe Melaku ICAP-Ethiopia, Country Director**

**Kirkos Kifle Ketema, Kebele 02/03, House#005**

**Africa Avenue (Bole Road), PO Box 5566**

**Addis Ababa, Ethiopia**

[**zy2115@columbia.edu**](mailto:zy2115@columbia.edu)

**+251 11 554 7460 (office)**

**+251 9 11 22 5347 (mobile)**

**Participation Duration:** 42 days

**Anticipated Number of Subjects:** 360

**Purpose**

The purpose of this research study is to find out how well the treatment for malaria is working in Ethiopia. This will help us to treat malaria better in the future. We are asking you to be part of this study because you have malaria. This study is supported by Federal Ministry of Health (FMOH), United States Agency for International Development, International Center for AIDS Care and Treatment Programs (ICAP) at Columbia University, and Centers for Disease Control and Prevention.

**Introduction**

We invite you or your child to take part in this study. It is important that you understand that taking part in this activity is entirely your choice and you may or may not experience personal benefits as a result. You may stop taking part at any time without penalty or loss of any benefits you are entitled to. Information from this study might help others. The purpose of the study, the benefits, risks, discomforts and other information are discussed below. You will be told of any new information that is discovered during the course of this study, which may affect your willingness to continue to be a part of the study. Up to 360 persons may be enrolled in this study. You will be followed closely for 42 days.

**Background**

Malaria is a sickness caused by a very small germ that can get into the body when a mosquito bites you. It can cause fevers, headaches, body aches, and weakness. If it is not treated, it can make a person very sick. When malaria is treated with the right drugs, it can be cured. Sometimes the germ that causes malaria can change and these drugs don’t work as well.

##### Procedures

Taking part in this study will last 42 days and will include nine visits to the clinic.

If you or your child agree to be in this study a staff person will check you or your child. At all but one of the nine visits, a small amount of blood will be taken from the tip of the finger (about 3 drops) after a needle prick. The blood will be checked for the malaria germ.

Each time we do a blood test for malaria, we will put a few drops of blood onto a piece of paper. This blood will be used to learn about the malaria germ that causes the illness. If the drug does not work as well as it should, some of this blood will be sent to a reference laboratory in Addis Ababa and the United States to check why the drug did not work. This blood sample will be destroyed once the tests are done and not stored.

Please be assured that if you decide that you or your child should not participate in this study, you or your child will be treated for malaria.

If you agree to participate or for your child to, you will get 1 of 2 drugs that are considered the first choice in Ethiopia depending on the type of malaria you or your child has. We will tell you which type of malaria was found in the blood.

- If you or your child has a certain kind of malaria (which doctors call *P. falciparum*) you or your child will always get artemether-lumefantrine (Coartem).
- If you or your child has the other type of malaria (which doctors call *P. vivax*) you or your child will get either artemether-lumefantrine (Coartem) or chloroquine. Both these drugs are normally given for this type of malaria and recommended in Ethiopia. This will be decided by chance (like the flip of the coin).

You or your child will need to come to the clinic to swallow the tablets every day for the first three days. We will watch you for about an hour after you take the medicine to make sure you do not vomit.

We will ask you or your child to return to the clinic 1, 2, 3, 7, 14, 21, 28, 35, and 42 days after the treatment starts so we can check to make sure that the malaria has been cured and does not return. We will give you a visit card so you know what days to come or have your child come.

At every visit, except tomorrow, we will do a finger prick blood test for malaria. A test to check for malaria in the blood will be done today and once each week. At each visit, a staff person will check you or your child and ask some questions. If you or your child does not come to clinic, someone from the clinic will come to your home to check on you.

We expect that these drugs will work well in most people. But if the malaria is not cured or if it comes back, we will provide treatment with another drug called quinine. This is the standard treatment in Ethiopia for this situation. This treatment should cure malaria. We will still keep checking to make sure that the malaria is fully cured.

If you or your child has a severe case of malaria, this will lead to admission to the hospital. There, strong drugs and other care will be given based on standards in Ethiopia.

###### Risks

There may be risks or discomforts if you take part in this study.

The drugs can cause an upset stomach, vomiting, diarrhea, headache, dizziness, mild skin rash, and itching. But these are mostly mild and soon go away.

Rarely, more severe side effects can occur. If you or your child has severe side effects, like persistent vomiting, severe rash, or dizziness and weakness with low blood pressure, we will stop the drug. We will also advise that you be treated with different malaria drugs in the future.

You or your child should come to the clinic right away if the sickness does not get better or it worsens and if there are any side effects from the drugs. You or your child can come back anytime even if not on a day you are scheduled to.

Make sure that you or your child come to clinic for appropriate care and possible transfer to a hospital if you have fever, convulsions, become very sleepy or cannot be woken up, have a lot of vomiting, are unable to drink or eat, have painful rash or mouth sores or red eyes, chest pain or difficult breathing or very fast breathing..

There are very little effects from the finger pricks like pain, bleeding, bruising, fainting, or infection. But these are very rare. We will clean the finger before taking blood and will use new needles to draw the blood each time.

There may be other risks of taking part in this study that we don't know about. If we learn about other risks, we will let you know what they are so that you can decide whether or not you or your child want to continue to be in the study.

###### Benefits

You may or may not get personal (direct) benefit from taking part in this study.

There are possible benefits of taking part in this study which follow:

You will not have to pay fees for any of the clinic visits during this study including any visits for other illnesses during the 42 days of follow up. You or your child will be closely followed for the next 42 days to see how well the drugs are working.

There will be someone here at the clinic every day. You may come for a visit at any time if you feel that you are ill, even on nights or weekends or in between visits.

This study will also help Ethiopia learn which drugs work best in this region. This may help you or someone you know in the future.

**Alternate Procedures**

If you decide that you do not want to take part or have your child be in this study, treatment for malaria will be provided.

###### Confidentiality

The results or information about you will be kept confidential to the extent allowed by law. Although every effort will be made to protect the privacy of the information about you or your child that is kept in the clinic, absolute privacy cannot be guaranteed. By signing this document you grant permission for information about you or your child obtained during the study to be made available to:

- The investigator, staff members, and other medical staff who may be evaluating the study.

- Staff from Columbia University and New York Presbyterian Hospital, the Center for Disease Control and Prevention, and the Ethiopian Ministry of Health, including the Institutional Review Board (IRB)/ ethical committees.

**Injuries**

Staff members will assist you in obtaining medical treatment, including emergency treatment, hospital care and follow-up care as needed. Any hospital stay which occurs during the 42 day follow-up period will be paid for. Compensation for injury that results from participation in this study is not available.

You do not give up any of your legal rights by signing this consent form.

**Compensation**

You will receive 35 Ethiopian Birr for each visit to pay for your travel to the clinic. If you are the caretaker accompanying a child over 6 years, you will receive 50 Birr.

**Additional Costs**

n/a

**Participation**

Taking part in this study is your choice. You can decide not to take part in or stop being in the study at any time. Your choice will not affect the treatment you receive for malaria. Also, none of the treatment you receive will be affected. You may leave the study at any time. This will not affect your health care, and you will still receive malaria treatment for free.

If a staff member needs to take you out of the study for any reason, then we will not continue to follow you. If you are removed from the study before the treatment is complete, or if the medicine did not make you better, then you will be referred to the clinic and treated with another treatment as noted in Ethiopia malaria recommendations.

**Additional Information**

Questions

If you have questions or concerns about taking part in this study, you may speak with one of the staff.

If you have any questions later about the study, please contact Dr. Zenebe Melaku at 09 11 22 5347 or Dr. Bereket HaileGiorgis at 011 554 74 54, or 09 11 40 5562.

If you would like to withdraw from the study, please contact Dr. Zenebe Melaku at 09 11 22 5347 or Dr. Bereket HaileGiorgis at 011 554 74 54, or 09 11 40 5562.

If you would like to speak with someone not directly involved with the study about your rights, please contact Dr. Daddi Jima at 091 140 5722 at the Ministry of Health.

**Statement of Consent**

By signing or placing my thumbprint below, I am saying that:

I have read this form, or it has been read to me; I have been able to ask questions about it, and my questions have been answered.

For adults: I understand that my or my child’s participation is voluntary and that I can leave the study at any time without it affecting my care.

For child aged 7-17 years: I understand that my participation is voluntary and that I can leave the study at any time without it affecting my care. My decision to participate is supported by my parent/ guardian but not forced by him/her.

For child aged 7-11 years: Read assent addendum below.

I agree to enter this study. I agree to report any unexpected or unusual symptoms.

I have received a copy of this form.

Signing this form does not waive any of my legal rights.

**For adult subjects**

Person Obtaining Consent

Print Name ____________________ Signature ________________ Date ________

Witness

Print Name ____________________ Signature ________________ Date ________

Subject

Print Name ____________________ Date _______

_

Thumbprint__________ Or Signature ________________________

**For subjects 6 months-17 years old**

Person Obtaining Consent

Print Name ____________________ Signature ________________ Date ________

Witness

Print Name ____________________ Signature ________________ Date ________

Subject

Print Name ____________________

Parent/Guardian

Print Name ____________________ Date ________

Thumbprint__________ Or Signature ________________________

**Assent Addendum for Children 7-11 years of age (Flesch-Kincaid Reading Level 3.1): (please read to 7-11 year olds)**

**Introduction**

The Federal Ministry of Health (FMOH), United States Agency for International Development, International Center for AIDS Care and Treatment Programs (ICAP) at Columbia University, and Centers for Disease Control and Prevention invite you to be in this research study.

**What is the purpose of the study?**

You have malaria. Malaria is a sickness that you can get when a mosquito bites you. The purpose of this study is to find out how well the drugs for malaria are working in Ethiopia.

**How long would you need me?**

You will come to the clinic nine times over the next 42 days.

##### What do you want me to do if I decide to be in the study?

If you agree to be in this study, a staff person will check you. We will take blood from your finger and ask you some questions each time you come. You will be given a drug to treat malaria depending on the type of malaria you have. You will need to come to the clinic to swallow the tablets every day for the first three days.

###### Are there any risks to me if I decide to be in the study?

There may be things you don’t like if you are in this study. The needle prick might hurt. The drugs can cause an upset stomach, headache, dizziness, or itching. But these go away soon.

If something more serious happens, your parent will bring you to the clinic where you will get treatment or be sent to the hospital.

###### Are there any benefits from being in the study?

You may or may not benefit from this study. You will not pay for anything during the 42 days. There will be someone here to see you everyday.

**Who should I call if I have any questions?**

If you have additional questions, ask your parent. They will know who you can call.

**Do I have to be in this study?**

Taking part in this study is your choice. You can decide not to take part in or stop being in the study at any time. Your choice will not affect your treatment for malaria.

### Assent: I was told about the study. I asked questions. I had my questions answered. I want to be in the study.

**For subjects 7-11 years old**

Person Obtaining Consent

Print Name ____________________

Signature ________________ Date ______________________

Witness

Print Name ____________________

Signature ________________ Date ______________________

Subject

### Print Name ____________________

### ANNEX VIII Data Safety Monitoring Board

1. **Data Safety and Monitoring Board (DSMB):**

The **Data Safety and Monitoring Board (DSMB)** is established based on the National Health Research Ethics Review Guideline 4th edition-June 2005; issued by the Health Department of the Ethiopian Science and Technology Council, with mandates to further protect the rights and welfare of the subjects in whom a medical intervention might increase the risk of adverse events that might occur and also assure the scientific integrity and validity of the findings.

- 1. **Objectives of the DSMB:**

The DSMB is established to protect the rights and welfare of the subjects and safeguard the safety of the ***study*** participants in whom a medical intervention might increase the risk of adverse events, and also assure the scientific integrity of the study as well as the validity of the study results.

- 1. **Accountability of DSMB:**

DSMB is accountable to ICAP Ethiopia and discharges its responsibilities through the Country Director of ICAP Ethiopia who is a designator of the DSMB.

- 1. **Responsibilities of the DSMB:**

**The responsibilities of the DSMB will be to:**

- Review research protocol, informed consent documents and plans for data safety and monitoring
- Evaluate the progress of the study, through periodic assessments of data quality and timeliness, participants recruitment, accrual and retention, participants risk versus benefit, performance of the trial site, and other factors that can affect study outcome;
- Consider factors external to the study when relevant information becomes available, such as scientific or therapeutic developments that may have an impact on the safety of the participants or the ethics of the trial;
- Review performance of the study sites, make recommendations and assist in the resolution of problems reported by the PI;
- Protect the safety of the study participants;
- Report on the safety and progress of the study to the appointing body;
- Ensure the confidentiality of the study data and the results of monitoring;
- Assist the designator by commenting on any problems with study conduct, enrolment, and sample size and/or data collection;
- Report the decisions to investigators who must submit those reports to the Institution Ethical Review Committee, which shall further report to NERC.
  1. **Composition of DSMB and eligibility criteria:**

The DSMB composed of experts external to the study group. The members of the Board:

- Are independent of the sponsor of the study and of the manufacturer of any product that is being evaluated
- Have no conflict of interest in the study they are monitoring
- Will receive no scientific recognition in the form publications or promotions from the results
- Are responsible enough to assume high level professional leadership
- Are recognized professionals representing the relevant expertise
- Have experiences in research undertakings and/or research management; and
- Are willing to undertake the responsibility

1. **Designated DSMB Members:**

Based on the eligibility criteria stated above, the following experts are selected as members of the DSMB for the study entitled “Assessing the efficacy of artemether-lumefantrine for the treatment of uncomplicated *P falciparum* infection and either artemether-lumefantrine or chloroquine for *P. vivax* infection”

| **Name** | **Qualification** | **Responsibility** |
| --- | --- | --- |
| Dr. Mengistu Tafesse | Paediatrician | DSMB Chairperson |
| Prof. Eyasu Mekonnen | Clinical Pharmacology | DSMB Member |
| Dr. Getahun Mengistu | Internist & Neurologist | DSMB Member |
| Dr. Alemayehu Worku | Bio-statistician | DSMB Member |
| Dr. Tsigereda Gadissa | Public Health Specialist | Administrator |

1. **Meeting arrangements:**

- The Secretary in consultation with the Chairperson calls regular and special meetings; and
- DSMB meets twice monthly, and special meetings will be convened any time as deemed necessary;

1. **Standard Operations Procedure (SOP) of DSMB:**

**The DSMB will have SOP that will be based on the following guidelines:**

- 1. **Membership criteria:** See eligibility criteria under 1.4.
  2. **Terms of appointment for DSMB**
- Members of the DSMB will serve for a minimum of six months
- A member that may resign will be replaced by the appointing body
  1. **Chairperson of the DSMB, duties and responsibilities:**
- Presides over the DSMC meeting; and
- Presents the proposed agenda to the Board for approval;
- Liaises with the DSMB appointing body and is responsible for all official communications regarding DSMB activities and reports
  1. **Administrator of the DSMB, duties and responsibilities:**

Administrator of the DSMB is a staff of ICAP that to be assigned by the Country Director of ICAP Ethiopia to support routine activities and implement the decisions of the Committee.

The responsibility of the Administrator will include;

- In consultation with the Chairperson, prepares and presents periodic DSMB activity plans;
- Undertakes a preliminary screening to ensure completeness of required documents;
- Organizes regular and special meetings in consultation with the chairperson;
- Facilitate the implementation of decisions by the DSMB;
- Keeps documents, relevant reports and minutes;
- Facilitates logistics and administrative support to DSMB;
- Establishes networking and facilitates communication with individuals and relevant offices; and
- Facilitates the preparation and timely submission of all regular reports
  1. **Procedure for reporting and addressing potential** or real conflict of interest
- All DSMB decisions signed by the Chairperson will be communicated to the PI through the Administrator
  1. **Quorum requirement:**
- The Quorum of meetings will be accepted when the majority (51%) of the Committee members are present
  1. **Meeting requirements and Minutes of the meeting:**

The following Procedures will be applicable for DSMB meetings:

- Decisions will be based on consensus basis. At times, decisions can be made by majority votes and all members have equal voting rights. At times when votes are at par, the position the chairperson takes will be final;
- Minutes shall be signed by those members who attended the DSMB meeting;
- The format of DSMB meeting will be close session unless the DSMB invites experts, one or group of investigators for clarification on the agenda;
- When a member is absent for three consecutive meetings without valid justifications, the Administrator in consultation with the Chairperson will request the Country Director of ICAP for replacement.
  1. **Review of the sponsors report:**
- All reports on the study or related to it will be reviewed by the DSMB.

1. **Recommendations:**

DSMB submits recommendations to the PI on the study and reports based on its roles and responsibilities. However, the sponsor is responsible for establishing a procedure for receiving and distributing the recommendations submitted by the DSMB.

1. **Documentation:**

- All minutes of meetings, communications and files shall be documented by the Administrator;
- All information and documents that reach to the DSMB Committee members shall be handled confidentially.
